# Functional Genomics Screening in *Chlamydomonas reinhardtii* Maps the Genetic Landscape of Tolerance to Paraquat and Diuron

**DOI:** 10.1021/acs.est.5c17308

**Published:** 2026-05-27

**Authors:** Tim Godec, Carissa Bleker, Katja Stare, Tjaša Lukan, Valentina Levak, Magda Tušek Žnidarič, Alexander Betz, Tina Kosjek, Katarina P van Midden, Marina Klemenčič, Francesco Trenti, Graziano Guella, Kristina Sepčić, Friedrich Fauser, Weronika Patena, Martin C. Jonikas, Maruša Kerenčič, Tina Eleršek, Mélanie Pietri, Thomas Rodet, Urban Bren, Marko Jukić, Samo Lešnik, Anže Županič

**Affiliations:** † 551837 National Institute of Biology, Department of Biotechnology and Systems Biology, Ljubljana 1000, Slovenia; ‡ 61790 Jožef Stefan Institute, Department of Environmental Sciences, Ljubljana 1000, Slovenia; § 112794 University of Ljubljana, Faculty of Chemistry and Chemical Technology, Ljubljana 1000, Slovenia; ∥ University of Trento, Department of Physics, Povo Trento 38123, Italy; ⊥ University of Ljubljana, Biotechnical Faculty, Ljubljana 1000, Slovenia; # 200547 Princeton University, Department of Molecular Biology, Princeton, New Jersey 08544, United States; ∇ Howard Hughes Medical Institute, Princeton, New Jersey 08544, United States; ○ National Institute of Biology, Department of Genetic Toxicology and Cancer Biology, Ljubljana 1000, Slovenia; ◆ Jožef Stefan International Postgraduate School, Ljubljana 1000, Slovenia; ¶ 28499 Eawag - Swiss federal institute of aquatic science and technology, Duebendorf 8600, Switzerland; ◘ University of Ljubljana, Faculty of Computer and Information Science, Ljubljana 1000, Slovenia; ▲ University Paris-Saclay, ENS Paris Saclay, CNRS, LuMIn, Gif-sur-Yvette 91190, France; ◮ University Paris-Saclay, CNRS, ENS Paris Saclay, LMF, Gif-sur-Yvette 91190, France; ■ 27048 University Paris-Saclay, ENS Paris Saclay, CNRS, Satie, Gif-sur-Yvette 91190, France; ⬠ 200917 University of Maribor, Faculty of Chemistry and Chemical Engineering, Laboratory of physical chemistry and chemical thermodynamics, Maribor 6000, Slovenia; ⬟ Faculty of Mathematics, Natural Sciences and Information Technologies, University of Primorska, Koper 6000, Slovenia; ⬤ Institute of Environmental Protection and Sensors, Maribor 2000, Slovenia

**Keywords:** functional genomics, Chlamydomonas reinhardtii, paraquat, diuron, ecotoxicology

## Abstract

Functional genomics offers a powerful, unbiased approach to elucidating the molecular mechanisms underlying the toxicity of environmental pollutants. In this study, we applied genome-wide screening in *Chlamydomonas reinhardtii* to investigate two classical herbicides: paraquat and diuron. Our screen successfully uncovered critical nuclear-encoded pathways that govern susceptibility. For both herbicides, we identified genes regulating the assembly and maintenance of the photosynthetic machinery, highlighting the central role of nuclear control over these chloroplast-localized targets. Beyond these target-related factors, we discovered novel nontarget-site resistance mechanisms. For paraquat, we identified intracellular trafficking as the central determinant of toxicity, experimentally characterizing a P5B ATPase transporter and a fatty acid elongation pathway whose disruption, we propose, converges on the same endomembrane delivery route through sphingolipid depletion. In contrast, our screening data suggest that diuron tolerance may be associated with a metabolic strategy focused on energy conservation, where the inactivation of specific NADPH-consuming enzymes could preserve reducing power for essential antioxidant defense. Collectively, these findings demonstrate that functional genomics can reveal novel, complex modes of action even for well-characterized chemicals, providing the mechanistic resolution required to advance modern ecotoxicological risk assessments.

## Graphical abstract

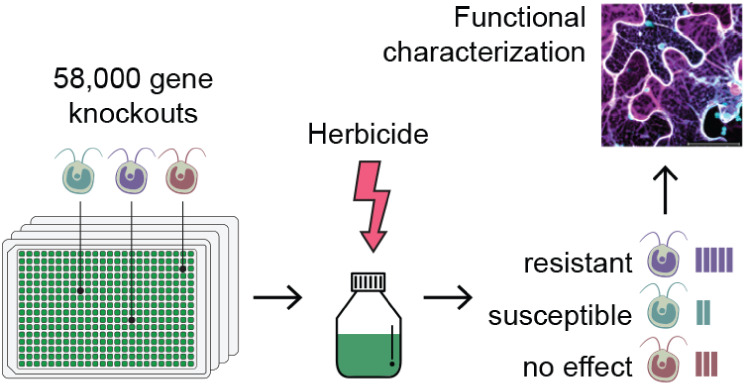

## Introduction

The widespread presence of synthetic chemicals in the environment requires robust methods for assessing their adverse effects on ecosystems and human health.^1^ Herbicides represent a major class of environmental contaminants, but traditional ecotoxicological tests that measure end points like survival or growth are often too low-throughput and mechanistically opaque for comprehensive risk assessment.^2,3^ While molecular techniques such as toxicogenomics can reveal global changes in gene expression, they cannot easily distinguish between functionally essential survival responses and those that are secondary or nonprotective. This makes it challenging to establish causal links between a specific gene and an organism’s fitness under chemical stress.^4^ Consequently, a key knowledge gap remains: identifying the genetic determinants of sensitivity and tolerance (defined as reduced or increased fitness under chemical exposure, respectively) is crucial for defining mechanisms of action (MoA) and developing predictive frameworks like Adverse Outcome Pathways (AOPs).^5^

Functional genomics can establish these causal links by directly testing the role of each gene in an organism’s survival under chemical stress.^6^ In a genome-wide screen, a library of mutants—each with a known gene inactivated—is exposed to a toxicant. The relative abundance of each mutant is then quantified. Mutants with inactivated genes that are essential for tolerance will be depleted from the population, whereas mutants lacking genes that mediate toxicity will become enriched. This “chemical-genetic fingerprinting” approach has been used successfully to uncover novel toxicity mechanisms in other model systems.^7–10^ With the recent development of a comprehensive mutant library for the green alga *Chlamydomonas reinhardtii*, this strategy can now be applied to a key photosynthetic primary producer.^11,12^ Its long-standing use as a standardized test species in regulatory guidelines and as a model organism for the study of photosynthesis makes it an ideal platform for such mechanistic investigations.^13–16^

Here, we apply this functional genomics strategy in *C. reinhardtii* to investigate the effects of two common herbicides: diuron and paraquat. Both compounds disrupt photosynthesis, but through distinct mechanisms.^17^ Diuron acts by blocking the electron transport chain within Photosystem II (PSII), which not only halts photosynthesis but also leads to the formation of highly reactive singlet oxygen (^1^O_2_), causing direct photo-oxidative damage to the reaction center.^18^ Paraquat, in contrast, intercepts electrons from Photosystem I (PSI), generating a continuous flux of superoxide radicals (O_2_^–^), which ultimately destroys the cell membranes.^19,20^ While their primary molecular targets are well-established, the full network of genes that governs cellular defense, detoxification, and overall fitness in response to these herbicides is not yet fully understood.

The goal of this study was, therefore, to systematically identify the genes in *C. reinhardtii* whose loss of function confers either sensitivity or tolerance to diuron and paraquat. By comparing the resulting chemogenetic profiles, we aimed to uncover both herbicide-specific and shared response pathways. Following this genome-wide screen, we selected key paraquat-tolerant candidate genes for in-depth experimental validation to characterize their specific roles in herbicide tolerance and toxicity.

## Methods

### Chemicals

Diuron and paraquat were purchased from Sigma-Aldrich. For the dose-finding and library screening experiments, diuron was dissolved in pure dimethyl sulfoxide (DMSO) to a concentration of 10 mg/mL. Subsequent dilutions were prepared with Tris-Acetate-Phosphate (TAP) medium to ensure that the final concentration of DMSO in the experimental cultures was maintained at or below 0.01% (v/v). Paraquat dichloride hydrate (Pq, Sigma-Aldrich) was dissolved in double-distilled water (ddH_2_O) to a concentration of 5 mg/mL, followed by serial dilution to achieve the desired treatment concentrations. For all validation experiments, diuron and paraquat were dissolved in Milli-Q water to a concentration of 5 or 10 μg/mL, with further dilutions made with TAP medium.

### Algal Strains and Culture Conditions

The wild-type (WT) *C. reinhardtii* strain CC-4533 served as the background strain for all of the experiments. The WT strain alone was used for preliminary dose–response experiments to determine the effective concentrations of diuron and paraquat. Additionally, two loss-of-function mutants (which showed the highest tolerance to paraquat) were obtained from the Chlamydomonas Resource Center (University of Minnesota, USA):

•LMJ.RY0402.155567 (mutant of Cre02.g093700)—used for paraquat uptake measurements, reduced growth validation, and electron microscopy

•LMJ.RY0402.062151 (mutant of Cre08.g373050)—used for lipidomics, reduced growth validation, and electron microscopy

All strains were grown in modified TAP medium^21^ under constant agitation (120 rpm), illumination of 100 μmol photon m^–2^ s^–1^, and a temperature of 22 °C.

### Dose–Response Experiments

#### Preliminary Dose-Finding

For the preliminary dose-finding experiments, WT was exposed to a range of diuron (20, 60, 180, and 540 nM) or paraquat (40, 120, 360, and 1080 nM) concentrations in 100 mL Erlenmeyer flasks containing 20 mL of culture. Cultures were grown for 72 h, with cell density measured at regular intervals using an automated CASY cell counter.

#### Mutant Validation

To validate the reduced growth phenotypes, the same setup was used, but the WT and the two mutant strains were exposed to paraquat at 50, 100, 200, 400, 800, 1600, and 3200 nM. Cell density was monitored for 72 h postexposure using the MACSQuant Analyzer 10 flow cytometer. Growth rates were calculated between 48 and 72 h postexposure, as this period was identified as the time when growth ceased for the highest concentrations. The resulting growth rates were compared between WT and mutant strains to confirm the phenotypes observed in the chemical screening.

### Mutant Library Description

For the chemical screenings, we used a genome-wide loss-of-function mutant library in *C. reinhardtii* developed by Li et al.^12^ in the CC-4533 strain. This library encompasses over 58,000 loss-of-function mutants, covering approximately 83% of annotated nuclear genes. Each mutant is identifiable by two unique barcodes on the 3′ and 5′ ends of a CIB1 insertion cassette. For detailed methodologies pertaining to mutant generation, insertion site mapping, and library maintenance, please refer to the original publications.^12,22^

### Chemical Screening and Sequencing

Chemical screening and barcode sequencing were executed as described previously^11^ (Figure 1a), with minor modifications. Briefly, individual mutants were pooled from 5-day-old 1,536-colony array plates, with the final cell density measured (Countess, Invitrogen) and adjusted to 1 × 10^5^ cells ml^–1^. Pooled cultures were inoculated at a density of 2 x10^4^ cell ml^–1^ and grown in 2-L bottles in modified TAP medium^21^ at 22 °C. Cultures were constantly mixed with a magnetic stirrer (200 rpm) and aerated under continuous illumination (100 μmol photon m^–2^ s^–1^).

**Figure 1:**
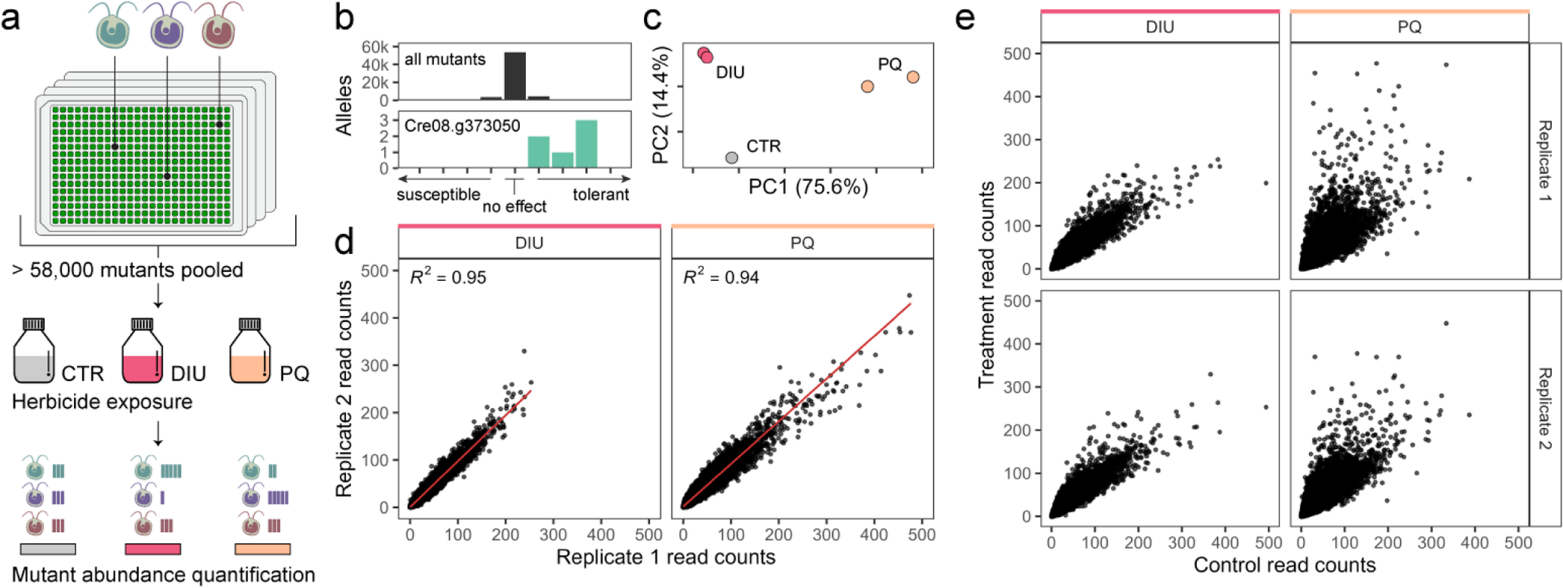
Overview of the functional genomics assay. **a)** Schematic overview of the pooled mutant screen. A library of >58,000 *C. reinhardtii* mutants was pooled and exposed to herbicide treatments (diuron, paraquat) or control conditions, followed by barcode sequencing to quantify mutant abundance. **b)** Distribution of mutant phenotypes under paraquat treatment used to determine high-confidence growth phenotypes: distribution of all mutants (top); distribution of alleles for gene Cre08.g373050 (bottom). **c)** Principal component analysis (PCA) showing clear separation between diuron (pink), paraquat (orange), and control (gray) samples. **d)** Correlation of barcode read counts between biological replicates for diuron and paraquat treatments (R^2^ = 0.95 and R^2^ = 0.94, respectively; counts >500 excluded for clarity, full data in Supplementary Figure 1). **e)** Comparison of mutant read counts between control and treatment samples for each replicate (counts >500 excluded for clarity, full data in Supplementry Figure 2). CTR: control conditions, DIU: diuron exposure, PQ: paraquat exposure.

Cultures were prepared in biological duplicates for each chemical treatment, with a single control. The treatments consisted of exposure to the determined EC20 concentrations of paraquat (42 μg/L) or diuron (19 μg/L), as determined from our preliminary dose–response experiments. After approximately 72 h (ca. seven cell divisions), cell density was monitored, and 2 × 10^8^ cells were harvested by centrifugation to form a frozen cell pellet.

The pellets underwent DNA extraction using the phenol/chloroform/isoamyl alcohol method. The resulting DNA was treated with RNase A, precipitated with ethanol, and then resuspended in water. The DNA concentration was measured using a Qubit fluorometer. The extracted DNA was used to amplify internal barcodes via PCR primers described previously.^12^ The PCR products were gel-purified, pooled, and sequenced on an Illumina HiSeq 2000.

### Identification of Growth Phenotypes

Reads containing barcodes were processed following the bioinformatic pipeline described in Fauser et al. (2022).^11^ Adapter sequences were removed using Cutadapt, and barcodes were counted by collapsing identical reads into unique sequences using fastx_collapser. Barcode counts of each experiment were normalized to a total of 100 million (Supplementary Table 1). For each mutant, the barcode with the highest normalized count in the control condition was used as the representative allele. Mutants were excluded from further analysis if any of the following criteria were met: any read count was equal to zero; the insertion was located in the 3′ UTR; the insertion position was unknown; or the likelihood of correct mapping was lower than 52% (see library description^12^).

Mutant susceptibility or tolerance to a treatment was assessed by comparing the abundance of each mutant after treatment to its abundance under control conditions. Mutant phenotypes were defined as the log_10_ ratio of normalized barcode counts between treatment and control samples (Supplementry Table 2). To determine high-confidence gene–phenotype relationships, we applied a statistical framework that aggregated mutant phenotypes across independent alleles. A gene’s representative phenotype was defined as the median phenotype of all alleles retained for testing. Each mutant was assigned to one of nine phenotype bins according to its log fold change: ←4; [−4, −3); [−3, −2); [−2, −1); [−1, 1); [1, 2); [2, 3); [3, 4); ≥ 4 (Supplementry Tables 3–4). For each gene, we constructed a contingency table reflecting the number of alleles in each bin (Figure 1B). Fisher’s exact test was used to assess enrichment relative to all insertions in the pooled replicates, and P-values were adjusted for multiple testing using the Benjamini–Hochberg procedure to control the false discovery rate (FDR). As an alternative to the method described to evaluate phenotype confidence, we have also developed a statistical method based on Gaussian Mixture Modeling (GMM). As the results of both methods were very similar, we only report Fisher’s results in the main text. The GMM method and phenotype calculations can be found in the Supplementary Methods and Supplementary Tables 3–4.

### Gene Set Enrichment Analysis

Enrichment of genes within functional groups was tested with gene set enrichment analysis (GSEA) using the fgsea package (v1.32.2)^23^ and ClusterProfiler^24^ (for KEGG pathways) in R. We selected six sets of gene annotations for testing: KEGG pathways, MapMan functional annotations, Gene Ontology terms (GO), GreenCut2 protein set, flagellar protein set, and protein localizations. ClusterProfiler^24^ was used for KEGG pathway enrichment analysis. MapMan functional annotations were predicted using Mercator4 (v6)^25^ via an online platform at https://www.plabipd.de/mercator_main.html, based on protein sequences from the *C. reinhardtii* CC4532 v6.1 annotation. Gene Ontology (GO) annotations were obtained from the PLAZA Dicots 5.0 database.^26^ GreenCut2 ^27^ protein IDs were converted from genome annotation v3 to v5.5 using the ID conversion tool at http://pathways.mcdb.ucla.edu/algal/id_conversion.html. The *C. reinhardtii* flagellar protein set^28^ was downloaded from https://chlamyfp.org/ChlamyFPv2/cr_read_sql.php. Protein localizations were taken from Wang et al.^29^

### Mutation Validation

To validate single mutants (**LMJ.RY0402.062151 and LMJ.RY0402.155567)**, we followed instructions for characterizing insertion sites by PCR, prepared by Ivanova and Zhang (https://www.chlamylibrary.org/files/Instructions%20on%20PCRs%20to%20check%20the%20insertion%20site.pdf) with some modifications: gDNA was isolated from single mutant cultures using the DNeasy Plant Mini Kit (Qiagen). PCR was performed with a Phusion Hot Start II HF Polymerase (Thermo Fisher Scientific).

Primers recommended for each mutant, and primers binding the CIB1 cassette, were obtained from https://chlamylibrary.org (Supplementary Table 5). The PCR products were purified (Wizard SV Gel and PCR Clean-Up Kit, Promega), and the sequence was determined by Sanger sequencing. The sequences were aligned to *C. reinhardtii* genes (Cre08.g373050 for mutant LMJ.RY0402.062151 and Cre02.g093700 for mutant LMJ.RY0402.155567) and to the CIB1 cassette to confirm the position of the insertion junction.

### DNA Extraction and Laddering

For DNA laddering assessment, 20 mL of cell suspensions were collected at 72 h after paraquat treatment, harvested by centrifugation (3 min, 3000 × g), and stored at −80 °C until further processing. DNA extraction was performed using the phenol/chloroform/isoamyl alcohol method according to the protocol of Tyler M. Wittkopp^30^ (see Supplementary Methods for details). Where possible, 6 μg of DNA was loaded onto a 1% agarose gel and visualized on a transilluminator.

### Localization Study

#### DNA Constructs

Two synthetic DNA fragments of the Cre02.g093700 gene, with a 30 bp overlapping part, were ordered from IDT (Integrated DNA Technologies). Fusion PCR was performed using the RepliQa HiFi ToughMix (Quantabio) to obtain a product of the entire gene. The *Cre02.g093700* gene was cloned into the GFP-containing pH7FWG2.0 vector or the RFP-containing pK7RWG2 vector (VIB) using the Gateway cloning system (Thermo Fisher Scientific) according to the manufacturer’s protocol. All constructs were verified by Sanger sequencing (Eurofins Genomics^31,32^ ).

The ER marker construct was generated by fusing a GFP sequence with an ER localization signal (KDEL; AAG GAC GAG CTG) at the C-terminal end, followed by a self-cleaving F2A peptide.^33^ The entire cassette was placed under the control of the CaMV 35S promoter and terminator. The construct was synthesized and assembled in a cloning vector pGH by ATG-Biosynthetics, flanked by I-SceI (NEB) restriction sites. Following restriction with I-SceI, according to provider’s instructions, the construct was purified using the Wizard SV Gel and PCR Clean-Up system (Promega) and inserted into the pCAMBIA_ ASX plant expression vector by NEBuilder HiFi DNA Assembly (NEB), following the protocol described previously.^34^

#### Agroinfiltration of *Nicotiana benthamiana*

Three-week-old *N. benthamiana* plants were used for transient transformation. Constructs were introduced into electrocompetent *Agrobacterium tumefaciens* GV3101 by electroporation, and effective transformation was confirmed by colony PCR (KAPA2G Robust HotStart, Kapa Biosystems), following our previously established procedure for *Agrobacterium* culture preparation.^34^ Cultures of the transformed cells were mixed with *Agrobacterium* transformed with an organelle marker (nucleus marker H2B protein tagged with red fluorescent protein (H2BRFP),^34^ ER marker (pCAMBIA_35S_GFP_KDEL_F2A; explained in the DNA constructs section), or plasma membrane (PM) marker^35^ and *Agrobacterium* transformed with the silencing suppressor p19 (kindly provided by Prof. Jacek Hennig) at a ratio of 1:1:2. The mixture was infiltrated into the first, second, and third fully developed bottom leaves of *N. benthamiana* plants as reported previously.^34^

#### Confocal Microscopy

The presence of the fused fluorescent proteins was visualized on the abaxial side of the detached, infiltrated *N. benthamiana* leaves with a Leica Stellaris 5 or Stellaris 8 confocal microscope. Images are presented as maximum projections from Z-stacks. Image overlays of all channel’s maximum projections from Z-stacks were performed using Leica LAS AF Lite software (Leica Microsystems). Detailed laser settings and acquisition parameters are provided in the Supplementary Methods.

### Electron Microscopy

Electron microscopy was performed on WT and both mutants. All cultures were grown in 100 mL Erlenmeyer flasks and were exposed to 1600 nM paraquat for 24 h. The cultures were fixed in 3% (v/v) glutaraldehyde diluted in 0.1 M PBS overnight. The next day, the pellet was mixed with 2% (w/v) agarose and cut into pieces up to 1 mm large in at least one direction. All samples were postfixed with 1% (w/v) osmium tetroxide and embedded in Agar 100 resin (Agar Scientific). Ultrathin sections were stained with an aqueous 1% (w/v) uranyl acetate and lead citrate. Ultrastructure was examined with a transmission electron microscope, Talos L120C (Thermo Scientific), operating at 100 kV. Micrographs were taken with a Ceta 16 M camera and edited in Velox software.

### Protein Structural Analysis

To characterize the predicted function of Cre02.g093700, an in silico structural analysis was performed by homology modeling using the hm_build protocol (v25.12.1), with the human polyamine transporter ATP13A2 (P5B-type ATPase; PDB ID: 7N75) identified as the top template. Independent homology models were constructed for eight conformational states of the transport cycle, and state-dependent molecular docking of paraquat and spermine was performed by using AutoDock Vina/Vinardo to assess binding site integrity and conformation-dependent substrate accessibility. The structural classification was further corroborated by AlphaFold3 modeling, Dali structural comparisons, and the identification of the conserved PP­(A/V)­L motif characteristic of P5B-type ATPases. Full details of the methodology, including sequence alignment, topology prediction, model quality metrics, and docking parameters, are provided in the Supporting Information.

### Intracellular Paraquat Measurements

Intracellular paraquat was measured in WT and the Cre02.g093700 mutant using the same culture conditions as for electron microscopy. The cells and the medium (supernatant) were separated by centrifugation (5 min, 1000 × g). The pellets were washed and lysed by three freeze–thaw cycles. Paraquat was extracted from cell lysates and analyzed by liquid chromatography coupled to tandem mass spectrometry (LC-MS/MS), as detailed in Supplementary Methods. The method’s limit of quantification (LOQ) was 5 ng/mL.

### Lipidomics

We prepared 400 mL cultures with 3–5 × 10^5^ cells/mL and left them to grow for 24 h. For lipidomic analysis, WT and Cre08.g373050 mutant strains were grown with and without paraquat (final concentration of 150 nM) for 24 h. After centrifugation (3 min, 3000 × g), algal pellets were collected, freeze-dried, and lipids were extracted according to Bligh and Dyer.^36^ Total lipid extracts were analyzed by liquid chromatography–mass spectrometry (LC-MS) coupled to a quadrupole ion-trap mass spectrometer equipped with an electrospray ionization source and operated in both positive and negative ion modes. Detailed chromatographic and mass spectrometry parameters are provided in Supplementary Methods.

### Statistical Analysis

Dose–response curves were fitted using the drc package in R,^37^ using the two-parameter log–logistic curve (function LL.2­()). For dose finding, the total growth rate until 72 h after exposure was taken as the relevant end point, while for the mutant validation experiments, the growth rate between 48 h and 72 h was taken as the end point, as this was the time at which the mutants and WT stopped growing at the highest paraquat concentrations. To compare intracellular paraquat concentrations between the WT and Cre02.g093700 mutant, we used Welch’s two-sample *t*-test.

To evaluate differences in lipid profile ratios between the wild type and the Cre08.g373050 mutant, we performed permutational multivariate analysis of variance (PERMANOVA) tests for each lipid class using Euclidean distance and 99,999 permutations. Lipid classes showing significant differences (*p* < 0.05) were further analyzed using univariate ANOVA on individual lipid species to identify the lipids contributing to the observed effects.

To assess the relationship between paraquat phenotypes and baseline growth rates, we obtained relative growth rates for each mutant from Supplementary Table 4 of Fauser et al. (2022),^11^ which used the same mutant library under identical growth conditions. Spearman’s rank correlation was computed between these relative growth rates and the paraquat phenotypes for all mutants retained in both data sets.

## Results and Discussion

### Overview of the Functional Genomics Screen

The functional genomics screen revealed distinct and highly reproducible cellular responses to paraquat and diuron. A principal component analysis (PCA) of mutant fitness profiles showed a clear separation between the two herbicide treatments and the untreated control, indicating unique chemical-genetic signatures for each compound (Figure 1C). Biological replicates for each treatment clustered tightly, confirmed by high correlation coefficients (R^2^ = 0.95 for diuron; R^2^ = 0.94 for paraquat), demonstrating the robustness of the experimental approach (Figure 1D, Supplementry Figure 1).

After applying stringent quality filters, we obtained growth phenotypes for 32686 mutants, representing 11089 genes (62.5% of the *C. reinhardtii* nuclear genome). The response to paraquat was markedly stronger than to diuron (Figure 1E, Supplementry Figure 2). At an FDR threshold of <0.3, in line with previous functional screens employing the same mutant library,^11,12^ we identified 98 genes whose loss of function significantly altered paraquat sensitivity. In contrast, only 74 such genes were identified under diuron exposure. This difference was even more pronounced for strong phenotypes (|logFC| > 1.5), with 18 hits for paraquat but only 3 for diuron (Table 1). This suggests that paraquat elicits a more widespread and severe cellular response compared to diuron.^19^

**Table 1:** Genes Showing the Strongest Mutant Phenotypes under Paraquat and Diuron Treatment^a^

Gene ID	Alleles	P-value	FDR	logFC	Phytozome 14 description	Phytozome 14 gene symbol	Phytozome 14 Auto Defline
Paraquat treatment							
Cre12.g548100	4	0.00040	0.110	–1.80	Putative Ubiquitin-protein ligase	UBC7	HECT-TYPE E3 UBIQUITIN TRANSFERASE
Cre16.g667150	4	0.00267	0.260	–1.77	/	/	Myc-type, basic helix–loop–helix(bHLH) domain
Cre03.g185200	10	0.00001	0.021	–1.60	protein ser/thr phosphatase	CPL3	SHEWANELLA-LIKE PROTEIN PHOSPHATASE 1
Cre12.g524300	4	0.00041	0.110	–1.58	conserved TPR repeat protein related to YCF37	CGL71	UDP-N-ACETYLGLUCOSAMINE–PEPTIDE N-ACETYLGLUCOSAMINYLTRANSFERASE 110 KDA SUBUNIT
Cre12.g486400	6	0.00019	0.081	1.55	/	/	PROTEIN TBC-3, ISOFORM B
Cre07.g342900	6	0.00148	0.205	1.57	/	/	S-methyl-5-thioribose kinase./MTR kinase.
Cre02.g090900	6	0.00059	0.128	1.59	Mitochondrial substrate carrier protein	MCP9	MITOCHONDRIAL SUBSTRATE CARRIER FAMILY PROTEIN J
Cre14.g614350	6	0.00004	0.041	1.63	/	/	DUAL ADAPTER FOR PHOSPHOTYROSINE AND 3-PHOSPHOTYROSINE AND 3-PHOSPHOINOSITIDE
Cre15.g641200	4	0.00007	0.045	1.72	Mitochondrial substrate carrier protein	/	solute carrier family 25 (mitochondrial uncoupling protein), member 8/9 (UCP2_3, SLC25A8_9)
Cre17.g706150	4	0.00057	0.126	1.76	/	/	/
Cre01.g014000	4	0.00065	0.135	1.78	/	/	/
Cre03.g167924	4	0.00006	0.044	1.90	/	/	very-long-chain (3R)-3-hydroxyacyl-CoA dehydratase./very-long-chain (3R)-3-hydroxyacyl-CoA dehydratase.
Cre02.g093700	14	0.00001	0.021	1.93	/	/	cation-transporting ATPase 13A3/4/5 (ATP13A3_4_5)
Cre01.g008850	6	0.00018	0.081	1.94	Histone-lysine *N*-methyltransferase	HLM2	Sec23-binding domain of Sec16 (Sec16_C)//Vesicle coat trafficking protein Sec16 midregion (Sec16)
Cre07.g324866	4	0.00012	0.067	1.99	/	/	TYROSINE-PROTEIN KINASE
Cre03.g149450	6	0.00002	0.031	2.21	/		Castor and Pollux, part of voltage-gated ion channel (Castor_Poll_mid)
Cre12.g554800	4	0.00002	0.031	2.61	Phosphoribulokinase, chloroplast precursor	PRK1	phosphoribulokinase./phosphopentokinase.
Cre08.g373050	6	0.00001	0.021	2.85	Acetyl-CoA biotin carboxyl carrier	BCC3	acetyl-CoA carboxylase/biotin carboxylase (ACAC)
Diuron treatment							
Cre06.g278148	4	0.00005	0.054	1.92	/	/	glyoxylate/succinic semialdehyde reductase (GLYR)
Cre06.g297400	4	0.00022	0.136	1.71	/	/	2-alkenal reductase [NAD(P)(+)]./NADPH:2-alkenal alpha,beta-hydrogenase
Cre12.g499400	4	0.00051	0.165	1.62	Cyclophilin	CYN18–2	peptidyl-prolyl cis–trans isomerase-like 1 (PPIL1)

a List of genes with significant changes in mutant growth (FDR < 0.3 and |median logFC| > 1.5) following herbicide exposure. For each gene, the number of independent mutant alleles, statistical significance (P-value, FDR), and median log fold-change in growth are shown. Phytozome curated gene symbols, descriptions, and computationally assigned functional descriptions (Auto Defline) are provided where available. Paraquat induced a larger number of strong phenotypes than diuron, consistent with the more pronounced global response observed Supplementry Tables 2–4.

### Most Sensitive and Tolerant Mutants

An examination of the 18 genes with the strongest paraquat-associated phenotypes (hereafter referred to as *top hits*; Table 1) provides a clear snapshot of the critical cellular processes governing its toxicity. The list is dominated by genes whose functions are directly linked to the integrity of the photosynthetic electron transport chain. The most sensitive mutants included those defective in Cre12.g524300 (CGL71), a well-characterized factor essential for protecting PSI from oxidative damage during its assembly,^38^ and Cre03.g185200 (CPL3), a metallophosphoesterase required for the stable accumulation of the Cytochrome *b*_6_f complex which links PSI and PSII.^39^ The essential role of both genes in photosynthesis was recently corroborated in a separate functional screen for photosynthetic phenotypes using the same mutant library.^40^ As paraquat’s toxicity stems from generating massive oxidative stress directly at PSI, the extreme sensitivity of these mutants logically follows: a cell already struggling to build or maintain the core photosynthetic machinery is acutely vulnerable to an agent that targets this very system. The other two sensitive hits include a putative E3 ubiquitin ligase (Cre12.g548100), likely involved in clearing damaged proteins, and Cre16.g667150, a gene (besides containing a Myc-type basic helix–loop–helix (bHLH) domain) of currently unknown function.

Conversely, the most tolerant phenotypes were consistently associated with genes involved in transport, lipid metabolism, and vesicle trafficking. The gene with the lowest P-value among tolerant mutants was a putative cation-transporting ATPase (Cre02.g093700). Given that paraquat is a dicationic molecule thought to mimic essential polyamines, the disruption of this transporter potentially confers tolerance by impairing the herbicide’s intracellular delivery to its chloroplast target. Another tolerant hit, Cre07.g342900, encodes a predicted S-methyl-5-thioribose kinase of the Yang (methionine salvage) cycle, which feeds into polyamine biosynthesis, providing a potential, though unconfirmed, second link between polyamine metabolism and paraquat toxicity. Three additional top hits encode core components of vesicle trafficking: Cre01.g008850 (HLM2), a predicted Sec16 vesicle coat protein involved in COPII-mediated ER exit; Cre12.g486400, a Rab-GAP (TBC domain) protein that regulates vesicle targeting; and Cre14.g614350, a pleckstrin homology (PH) domain protein predicted to bind phosphoinositides,^41^ which are key regulators of membrane identity and vesicle sorting—together pointing to the same process of intracellular paraquat delivery. The gene with the strongest phenotype (highest logFC) was BCC3 (Cre08.g373050), the cytosolic/ER-associated homomeric acetyl-CoA carboxylase that supplies malonyl-CoA for extraplastidial fatty acid elongation.^42,43^ Another lipid biosynthesis gene among the top hits was a 3-hydroxyacyl-CoA dehydratase (Cre03.g167924), which catalyzes a downstream step in very-long-chain fatty acid (VLCFA) synthesis in the ER. Two potential explanations exist for how disrupting extraplastidial fatty acid metabolism confers tolerance: (1) since VLCFAs are essential precursors of sphingolipids, which are in turn required for vesicle tethering, docking, and fusion in yeast and plants,^44–47^ their depletion may impair endomembrane trafficking, affecting paraquat delivery; or (2) slowing the synthesis of essential membrane components may induce a slower-growing metabolic state that reduces the overall photosynthetic electron flow fueling paraquat’s toxicity. The latter hypothesis is supported by another top hit, the phosphoribulokinase (Cre12.g554800, PRK1), an enzyme essential for regenerating RuBisCO in the Calvin cycle. Its absence would halt carbon fixation, causing a depletion of NADP^+^, the final electron acceptor for PSI, which would slow down the electron flow and thus give paraquat less opportunity to generate ROS. The remaining tolerant hits include two mitochondrial substrate carriers (Cre15.g641200 and Cre02.g090900 (MCP9)), a predicted voltage-gated ion channel (Cre03.g149450, Castor/Pollux family), and a predicted tyrosine-protein kinase (Cre07.g324866). The last two tolerant top hits (Cre17.g706150, Cre01.g014000) currently have no functional annotation available (Table 1). At a more stringent FDR <0.1 threshold, 11 of the 18 hits remain significant (10 tolerant, 1 sensitive); notably, both experimentally characterized genes (Cre02.g093700 and Cre08.g373050) and all core trafficking hits are retained, while the genes lost include CGL71 and Cre12.g548100 among sensitive hits and four tolerant genes with less defined roles (Cre07.g342900, MCP9, Cre17.g706150, Cre01.g014000).

The genetic signature of diuron tolerance was functionally distinct and pointed toward a strategy of conserving reducing power. The top hits included Cre06.g278148 (predicted glyoxylate reductase) and Cre06.g297400 (predicted alkenal reductase), both of which are enzymes that consume large amounts of NADPH. Diuron blocks PSII, starving the cell of new ATP and NADPH. Under this stress, we hypothesize that the photorespiratory cycle (where glyoxylate reductase functions) and the detoxification of secondary oxidative damage (where alkenal reductase functions) can potentially become expensive pathways; therefore, their disruption could lead to more reducing power being available for antioxidant defense. However, this proposed mechanism remains to be validated experimentally.

### Pathways Governing Paraquat and Diuron Tolerance and Susceptibility

To translate gene lists into biological mechanisms, we performed a gene set enrichment analysis (GSEA). The results pinpointed both expected and novel pathways, revealing a complex interplay between photosynthesis, vesicle trafficking, and metabolism in determining herbicide fate. The top-ranked enrichments for each herbicide are shown in Figures 2 and 3 for paraquat and diuron, respectively, with full results provided in Supplementary Tables 6 and 7.

**Figure 2:**
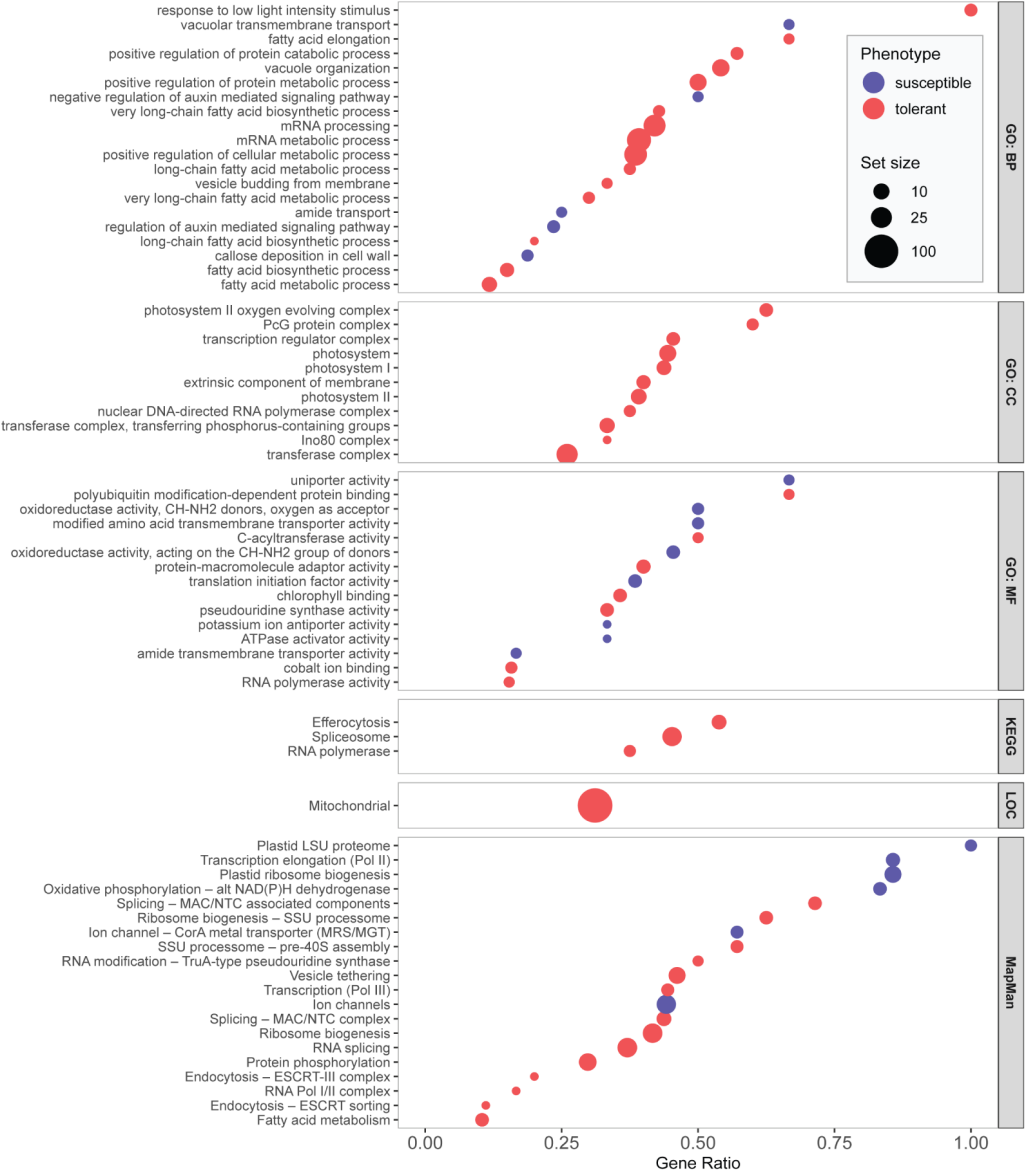
Enriched functional categories in response to paraquat. The *x*-axis shows the gene ratio; the *y*-axis lists functional terms; dot size indicates gene set size; dot color reflects enrichment direction (red = tolerant, blue = susceptible). For GO and MapMan annotations, only gene sets with 3–100 members and a P-value below 0.03 were considered. For KEGG pathways, localization, and GreenCut2 sets, a P-value cutoff of 0.05 was used with no size restriction. Up to 20 top-ranking terms per annotation set were plotted. Terms related to multicellular organisms’ processes and redundant terms were removed.

**Figure 3:**
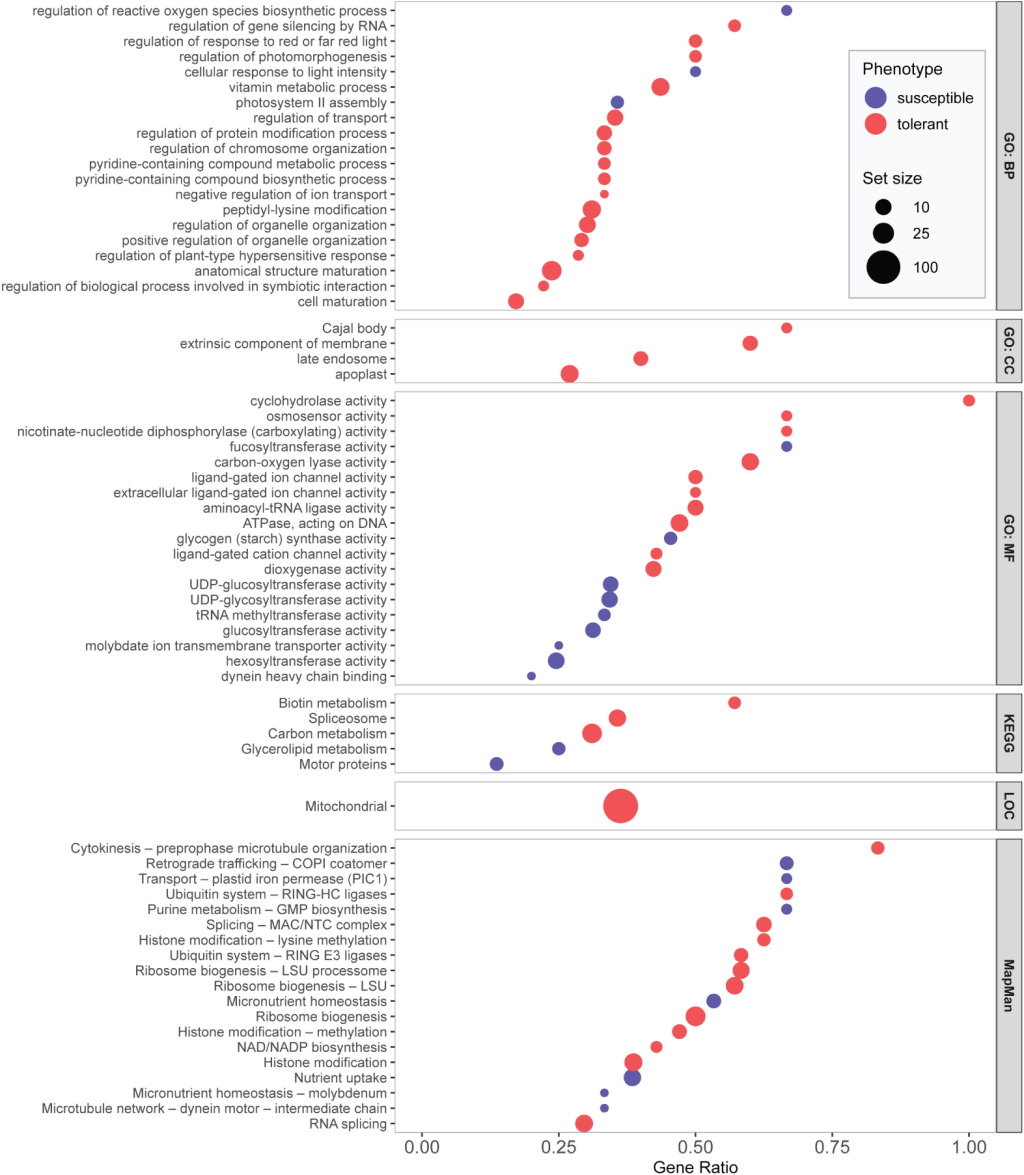
Enriched functional categories in response to diuron. The *x*-axis shows the gene ratio; the *y*-axis lists functional terms; dot size indicates gene set size; dot color reflects enrichment direction (red = tolerant, blue = susceptible). For GO and MapMan annotations, only gene sets with 3–100 members and a P-value below 0.03 were considered. For KEGG pathways, localization, and GreenCut2 sets, a P-value cutoff of 0.05 was used with no size restriction. Up to 20 top-ranking terms per annotation set were plotted. Terms related to multicellular organisms’ processes and redundant terms were removed.

### Photosynthesis-Related Responses to Paraquat and Diuron

For both herbicides, enrichment patterns were aligned with their known targets in photosynthesis. Gene sets related to the assembly, repair, and translation of photosynthetic components were associated with either susceptibility or tolerance, emphasizing the central role of photosynthetic processes in the cellular response to these herbicides.

In the case of paraquat, susceptibility was primarily linked to plastidial ribosome biogenesis, including genes encoding chloroplast ribosomal proteins (L18, L22, L34) and RAP-domain RNA-binding proteins required for rRNA processing. As plastidial ribosomes are necessary for the translation of plastid-encoded subunits of the photosynthetic electron transport chain, disruption of this machinery may impair the synthesis or repair of damaged photosystems. Conversely, tolerance was associated with mutations in chlorophyll-binding proteins and PSI and PSII light-harvesting proteins (LHCA2, LHCA3, LHCB-type proteins). Reduced function of these antenna complexes and oxygen-evolving components could limit electron flow to paraquat, potentially lowering the generation of reactive oxygen species (ROS) and thereby lessening toxicity. These results suggest that, while the photosynthetic machinery enables paraquat’s activity, the loss of certain light-harvesting components might mitigate its damaging effects.

For diuron, all photosynthesis-related enrichments were associated with susceptibility. Gene sets involved in PSII assembly and repair (e.g., PSB27-H2, LPA3, and other PSII biogenesis factors), thylakoid organization, and chloroplast iron homeostasis via the plastidial permease PIC1 were overrepresented among the sensitive mutants. Given that diuron inhibits electron transfer at PSII, these enrichment patterns are consistent with a scenario where efficient PSII assembly and repair help to maintain limited photosynthetic activity under stress, whereas impairment of these processes increases vulnerability.

Overall, the results support the expected connection between herbicide action and photosynthetic function. The opposing trends observed for paraquat and diuron, where the loss of photosystem components enhances tolerance to paraquat but increases sensitivity to diuron, point to distinct dependencies on photosynthetic electron flow for each compound’s toxic effect.

### Paraquat-Specific Response Patterns

Beyond photosynthesis, paraquat tolerance was associated with gene sets involved in vesicle trafficking and vacuolar organization. Enriched terms included components of the ESCRT and CORVET complexes, SNARE proteins, and other regulators of endosomal and vacuolar transport. Several MapMan and GO categories pointed to processes such as vesicle tethering, endocytic trafficking, and vesicle budding, with genes encoding Rab-type GTPases, vesicle coat proteins, and vacuolar fusion factors (e.g., MON1, VPS16, VAMP72). One potential explanation is the disruption of paraquat’s intracellular delivery route to the chloroplast. In *Arabidopsis*, the Golgi-localized transporter PAR1/AtLAT4 (no homolog exists in *C. reinhardtii*), whose disruption confers a tenfold increase in paraquat tolerance, has been suggested to use a vesicle-based system to transport paraquat toward its site of action;^48^ therefore, crippling this trafficking machinery would reduce the toxin’s concentration where it is most damaging. This hypothesis is additionally supported by findings in goosegrass, where the upregulation of SYP121 (a SNARE protein) expression also leads to paraquat tolerance.^49^ Consistent with this, individual genes within the inositol phosphate metabolism pathway showed a directional split: mutants of enzymes producing endosome-associated phosphoinositides (PtdIns3P) and soluble inositol phosphates (IP_3_, IP_4_, IP_5_) conferred tolerance, while those required for myo-inositol and phosphatidylinositol biosynthesis conferred susceptibility (Supplementry Figure 3), directly linking phosphoinositide-dependent endosomal identity to paraquat tolerance. An alternative explanation involves the classic growth-defense trade-off: disrupting a fundamental process like vesicle trafficking forces the cell into a slower metabolic state, which would reduce the photosynthetic electron flow that fuels paraquat’s toxicity.

Another pattern observed among paraquat-tolerant mutants involved very long-chain fatty acid (VLCFA) and sphingolipid biosynthesis. Apart from the already mentioned Cre08.g373050 and Cre03.g167924, there was an enrichment of genes linked to ceramide synthesis (e.g., Cre09.g400516) and related lipid metabolic steps. Since VLCFAs and ceramides are important components of membranes and signaling molecules, changes in their biosynthesis could influence how cells respond to oxidative stress. For instance, ceramides can participate in stress-induced cell death signaling; therefore, we hypothesize that reduced flux through this pathway might shift the balance toward survival under paraquat exposure.

Finally, transcription and RNA splicing emerged as a third theme. Tolerant mutants were enriched for genes involved in pre-mRNA splicing (e.g., the spliceosome and MAC/NTC complexes). These findings suggest that the loss of core splicing components may shift alternative splicing patterns in a way that enhances tolerance, as has been shown in plants,^50^ potentially by reducing ROS-induced damage. The involvement of TFIIH, which also participates in nucleotide excision repair, further hints at a link between oxidative stress and DNA damage. As with vesicle trafficking, disrupting such a fundamental process may also confer tolerance via the growth-defense trade-off, slowing overall metabolism and thus reducing the herbicide’s efficacy.

### Diuron-Specific Response Patterns

Beyond the photosynthetic susceptibility, diuron tolerance showed no clear secondary pattern. Most enriched terms were scattered across unrelated processes, with 16 of the 20 top GO biological process terms referring to higher plant developmental pathways (e.g., carpel, gynoecium, trichoblast, or embryo sac development), reflecting annotation bias rather than true functional relevance in *C. reinhardtii*. Among the localization sets, mitochondrial proteins were modestly enriched (FDR = 0.0354), possibly hinting at effects on oxidative stress or energy metabolism.

Overall, the lack of a dominant cellular signature suggests that the genetic basis for diuron tolerance is fundamentally different from that of paraquat. While paraquat tolerance was experimentally shown to involve broad, system-wide remodeling (lipidome alteration confirmed by lipidomics; vesicle trafficking suggested by GSEA), the genetic signature of diuron tolerance may depend on discrete metabolic adjustments—such as the inactivation of specific NADPH-consuming enzymes identified among our top hits—which do not manifest as broad pathway enrichments in GSEA. This proposed mechanism requires experimental validation through individual mutant characterization and direct biochemical measurements.

### Comparison to Known Mechanisms of Herbicide Resistance and Defense

Our functional genomics screen in *C. reinhardtii* was designed to systematically identify nuclear-encoded genes affecting herbicide tolerance (as defined in the introduction). For both herbicides, the primary target protein is encoded in the chloroplast genome (PsbA for diuron and PSI components for paraquat), meaning that our nuclear mutant screen cannot capture target-site resistance (TSR). It is, however, well-suited to uncover two other major classes of mechanisms within the agronomic resistance framework: 1) non target-site resistance (NTSR) pathways that regulate the availability of the herbicide, and 2) nuclear genes that control the expression, assembly, and repair of the chloroplast-encoded target.

For paraquat, for which no TSR has been found, our results align remarkably well with the major NTSR mechanisms involving impaired transport and delivery.^51^ First, our top-ranking individual-tolerant mutant is in Cre02.g093700, a gene encoding a putative cation-transporting ATPase. Second, our GSEA results independently identified vesicle trafficking and vacuolar organization as a major tolerance-associated pathway, supported by individual hits in genes such as the Rab-GAP protein Cre12.g486400. Interestingly, while our top hit is a putative transporter, it is not a homolog of the specific polyamine transporters previously identified in higher plants.^52–54^ This, combined with the fact that our screen also did not identify known efflux transporters^55^ or ROS detoxification genes^56,57^ as top hits, suggests that *C. reinhardtii* relies on a distinct and novel set of NTSR mechanisms to combat paraquat toxicity.

For diuron, beyond these target-related pathways described above, our screen suggests that disrupting NADPH-consuming enzymes may represent a potential NTSR strategy. We were not able to find many genes involved in herbicide metabolism, which has so far been the primary NTSR found in resistant weeds. This suggests that the genetic architecture of paraquat and diuron tolerance in *C. reinhardtii* is distinct from that of higher plants, but it is also worth noting that our loss-of-function screen is not designed to detect any gain-of-function resistance mechanisms observed in plant herbicide resistance.

A comparison of our genetic screen with a previous proteomic analysis^58^ of diuron/paraquat-exposed *C. reinhardtii* reveals very little overlap. This finding, while perhaps surprising, is consistent with numerous studies^59^ that have observed a lack of correlation between genetic and proteomic or transcriptomic responses. This discrepancy arises because the two approaches ask fundamentally different questions. A proteomics study, like that of Nestler et al.,^58^ reveals the cell’s dynamic adaptive response. For instance, they found that paraquat exposure led to the strong upregulation of antioxidant enzymes (like glutathione S-transferases and ascorbate peroxidase 1) and protein degradation machinery. For diuron, they observed an accumulation of the D1 protein (PsbA) and the upregulation of specific chloroplast antioxidants, reflecting a targeted stress response. In contrast, our functional genomics screen identifies the nonredundant genes whose absence fundamentally alters tolerance. This reveals the core machinery that mediates toxicity (like the vesicle trafficking system) rather than the downstream, often redundant, proteins involved in the “cleanup” response.

### Experimental Validation and Characterization of Top Paraquat-Tolerant Mutants

To validate the top hits from the screen, we selected the putative transporter (Cre02.g093700, lowest P-value) and the acetyl-CoA carboxylase (Cre08.g373050, highest phenotype) for further characterization. We first confirmed the cassette insertion sites in both mutants (LMJ.RY0402.155567 and LMJ.RY0402.062151) via PCR and Sanger sequencing, verifying them as true loss-of-function alleles.

We then confirmed their phenotypes and baseline growth characteristics. Under normal control conditions, both mutants exhibited a significantly slower specific growth rate than the WT. The WT specific growth rate was 0.064 ± 0.0001/h, compared to 0.047 ± 0.001/h for the Cre08.g373050 mutant and 0.042 ± 0.001/h for the Cre02.g093700 mutant (raw data available in Supplementary Table S8). At the same time, both mutants exhibited significantly higher tolerance to paraquat compared to the WT strain, with their growth curves clearly shifted to the right, confirming the protective effect of these gene knockouts (Figure 5a). These findings validate the results of the primary screen and confirm these genes as critical determinants of paraquat sensitivity; however, it does not provide a definite answer to the mechanism question, as the results are consistent with both a specific paraquat toxicity mechanism and the “growth-defense trade-off” hypothesis discussed in the GSEA section, which suggests that their reduced metabolic and photosynthetic activity may inherently contribute to the tolerance.

Two lines of evidence argue against slow growth as the primary explanation. First, the Spearman rank correlation between paraquat phenotypes and baseline growth rates across the entire library (obtained under identical conditions; Fauser et al.^11^) was significant but very weak (ρ = −0.13, *p* < 10^–125^), with growth rate explaining only 1.8% of the variance in paraquat tolerance (Suppl. Figure 4). Second, neither Cre02.g093700 nor Cre08.g373050 appeared as high-confidence hits (FDR < 0.3) under any other ROS-generating condition in the same library screen (hydrogen peroxide, Rose Bengal, UV irradiation, high light; Fauser et al.^11^). Together, this comparative evidence indicates that the tolerance conferred by these mutants is largely independent of growth rate and specific to paraquat rather than reflecting a generic antioxidant response.

### Characterization of the Putative Transporter Cre02.g093700

Having validated its strong phenotype, we sought to understand the mechanism by which the Cre02.g093700 transporter mediates paraquat toxicity. We first determined the protein’s subcellular localization by expressing a fluorescently tagged version in *N. benthamiana* leaves (attempts to do this in *C. reinhardtii* failed). Confocal microscopy revealed that the protein did not colocalize with the chloroplast or the nucleus (Figure 4, panel c). Instead, it showed clear colocalization with an endoplasmic reticulum (ER) marker (Figure 4, panel a) and with the plasma membrane (PM) marker (Figure 4, panel b). In addition to the ER and PM signal, punctate structures were observed that are consistent with transport vesicles trafficking between these compartments, though confirmation would require additional markers for vesicles. This localization to the cell’s secretory and outer boundaries is fully consistent with its predicted function as a membrane transporter and the recent computationally predicted localization of the protein in *C. reinhardtii*,^29^ placing it in an ideal position to mediate the internal transport to the chloroplast target.

**Figure 4:**
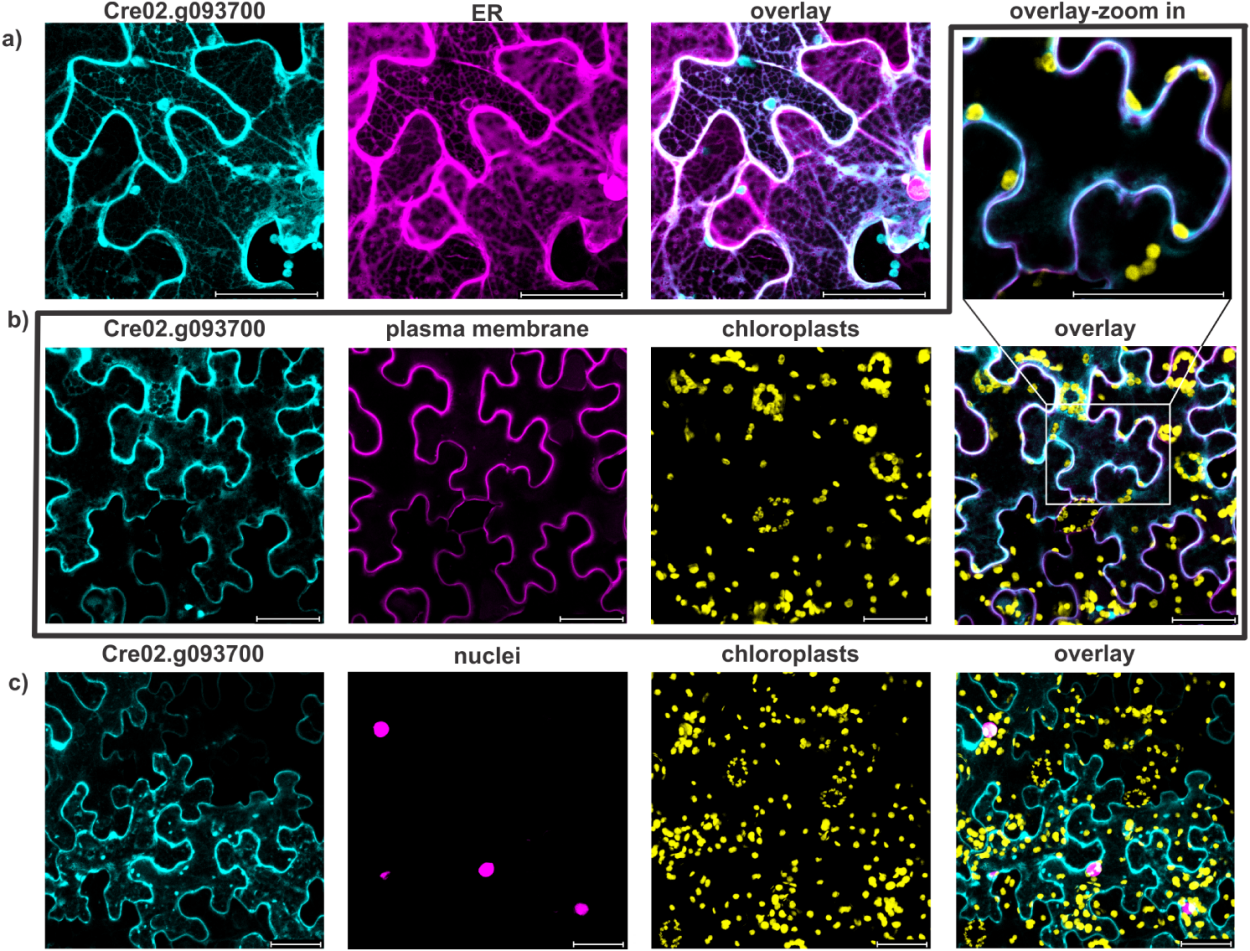
Localization of Cre02.g093700 protein. Representative confocal microscopy images of *N. benthamiana* epidermal cells showing: a) Cre02.g093700-RFP (represented as cyan), ER marker-GFP (represented as magenta), and overlay. b) Cre02.g093700-GFP (represented as cyan), PM–mCherry marker (represented as magenta), chlorophyll autofluorescence in chloroplasts (represented as yellow), and overlay. c) Cre02.g093700-GFP (represented as cyan), nucleus marker H2B-RFP (represented as magenta), chlorophyll autofluorescence in chloroplasts (represented as yellow), and overlay. White color (overlay channel) confirms colocalization of GFP and RFP/mCherry. Bar: 50 μm.

To determine the molecular identity of Cre02.g093700, we first generated a structural prediction using AlphaFold3 and compared it against the Protein Data Bank using the DALI server.^60^ The top structural matches correspond exclusively to conformers of human ATP13A2. To validate this classification, we performed extensive homology modeling using experimental cryo-EM structures of human ATP13A2 as templates, representing eight conformational states of the transport cycle (Supporting Information). Convergent template selection, robust model quality metrics, identification of the conserved PP­(A/V)­L motif, and preservation of characteristic P-type ATPase topology consistently supported the classification of Cre02.g093700 as a P5B-type ATPase orthologous to human ATP13A2, a well-characterized lysosomal polyamine exporter.^61–63^ The predicted structure contains ten transmembrane helices and the P5B-specific domain architecture characteristic of this subfamily. Notably, P5B ATPases have been lost in the flowering plant lineage but are retained in green algae,^64^ indicating that Cre02.g093700 represents an ancestral polyamine transporter. This identification is directly relevant to paraquat toxicity, as paraquat is a dication that structurally mimics the natural polyamine substrates (putrescine, spermidine, and spermine) of P5B ATPases. In mammalian cells, ATP13A2 expression specifically enhances paraquat toxicity but not sensitivity to other oxidative stressors.^61,65^

To test whether the protein retains a functionally intact binding site compatible with paraquat, we performed state-dependent molecular docking of paraquat and spermine (positive control) across all eight modeled conformations (Supporting Information Figure 5). Neither compound occupied the expected luminal substrate cavity in E1- or E1P-like states, whereas both consistently populated the binding site in all E2-family conformations (Supporting Information Figure 6). This state-dependent accessibility is characteristic of the known P5B transport mechanism and supports the functional assignment of Cre02.g093700 as a polyamine transporter with a binding site that is structurally compatible with paraquat.

To test whether the transporter affects paraquat accumulation, we quantified total intracellular paraquat levels in mutant and WT using LC-MS/MS. We detected no significant difference between strains (WT: 35.26 ± 6.70 ng/10^8^ cells; Cre02.g093700 mutant: 30.85 ± 6.04 ng/10^8^ cells; p = 0.52; Welch *t*-test). The absence of a difference in total cellular paraquat is consistent with the protein mediating intracellular redistribution rather than uptake. To directly confirm the redistribution model, subcellular fractionation experiments would be required.

### Characterization of the Lipid Synthesis Gene Cre08.g373050

We next investigated the Cre08.g373050 (BCC3) mutant, which lacks the cytosolic acetyl-CoA carboxylase, hypothesizing that the resulting disruption of the cytosolic malonyl-CoA pool constitutively alters extraplastidial lipid metabolism. A comprehensive lipidomic analysis was performed to test this hypothesis. We analyzed all major lipid classes from samples grown under both control and paraquat-exposed conditions. This analysis revealed that the mutant’s lipid profile was constitutively different from the WT, as paraquat exposure itself did not induce significant changes in either strain (data not shown).

We found no significant differences in either galactolipid, the main constituent of the thylakoid membrane, or sphingolipid, which was below our detection limit in all samples. Unchanged galactolipids indicate that plastidial lipid biosynthesis remains unaffected. The most significant changes were observed in storage lipids and extra-plastidial membrane lipids (Figure 5b; full results in Supplementary Figure 7). Compared to WT, the mutant showed an increased abundance of a free fatty acid (FFA 18:1), consistent with reduced consumption of the FFA pool due to impaired VLCFA elongation, alongside shifts in the relative abundance of individual triacylglycerol (TAG) and DGTS (diacylglyceryl-N,N,N-trimethylhomoserine) species. These alterations are confined to ER-derived lipid classes, supporting a cytosolic/ER-localized role for BCC3. Together, the data indicate constitutive remodeling of extraplastidial lipids, while chloroplast membranes remain unaffected.

**Figure 5:**
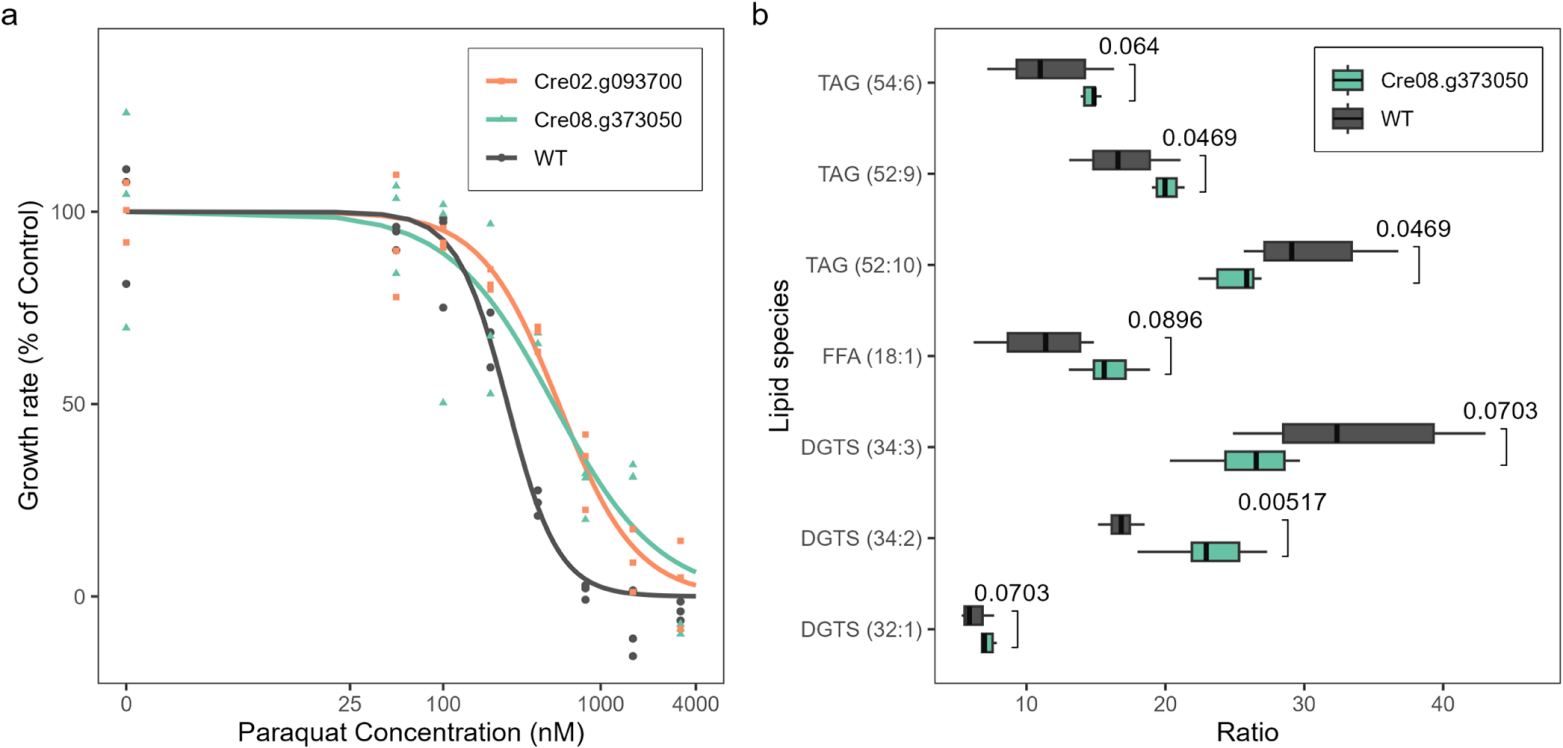
Validation of paraquat-tolerant mutants and lipidomic alterations in the acetyl-CoA carboxylase mutant. a) Dose–response growth curves of wild-type (WT), Cre02.g093700 (putative transporter), and Cre08.g373050 (acetyl-CoA carboxylase) mutants under paraquat exposure. Lines show fitted log–logistic models; points represent individual replicates. Both mutants display increased paraquat tolerance compared to WT. b) Lipid species significantly differing (FDR-adjusted *p* < 0.1) between WT and Cre08.g373050. The mutant shows altered triacylglycerol (TAG), free fatty acid (FFA 18:1), and DGTS profiles, consistent with disruption of the cytosolic malonyl-CoA pool and impaired extraplastidial lipid assembly.

We next investigated the physical and biochemical effects of the mutant exposed to a high concentration of paraquat. Using transmission electron microscopy (TEM), we did not detect large differences in ultrastructure between WT and mutant under control conditions (Figure 6a vs b). However, after exposure to 1600 nM paraquat, the WT cells exhibited a necrotic-like collapse. These changes included diminished electron density of the whole cell, extension of the plasmalemma toward the cell wall, collapse of the chloroplast—specifically loss of integrity of thylakoids and pyrenoid organization and its electron density—lower content of starch, disintegrated eyespot, dilatation of mitochondria, and detachment of flagella. This visual evidence of necrotic collapse is consistent with our biochemical findings. DNA laddering assays performed on both WT and mutant strains showed no evidence of the characteristic DNA fragmentation associated with apoptosis (Supporting Information Figure 8). Together, these results define the mode of paraquat-induced cell death in *C. reinhardtii* as a nonapoptotic, necrotic process. In striking contrast to the WT, the Cre08.g373050 mutant cells were completely protected from this fate. Their ultrastructure was remarkably preserved and appeared indistinguishable from that of the untreated control cells (Figure 6d).

**Figure 6:**
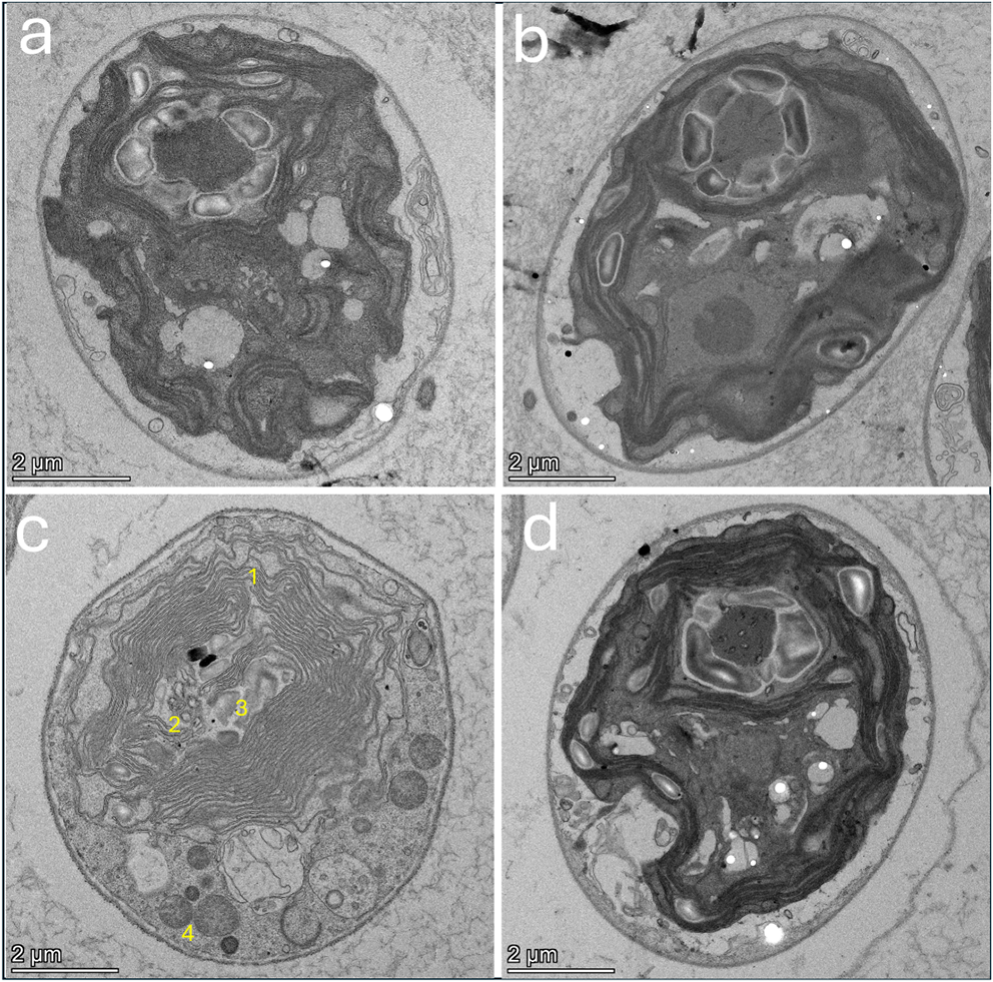
*C. reinhardtii* phenotypes in control conditions and treated with paraquat. Wild type (a), Cre08.g373050 mutant (b), wild type treated with 1600 nM paraquat (c), and Cre08.g373050 mutant treated with 1600 nM paraquat (d). Note the diminished electron density of the whole cell, extension of the plasmalemma toward the cell wall, collapse of the chloroplast—loss of integrity of thylakoids (1) and pyrenoid organization (2), lower content of starch (3), and dilatation of mitochondria (4) in wild type (c), while the Cre08.g373050 mutant showed a preserved phenotype despite treatment with a high concentration of paraquat (d).

### Proposed Mechanisms of Tolerance

Our data point to intracellular trafficking as the central process governing paraquat tolerance. Rather than two independent mechanisms, we propose a model in which paraquat hijacks the endomembrane system to reach its chloroplast target, and disruption of this system at multiple levels confers tolerance (Figure 7).

**Figure 7:**
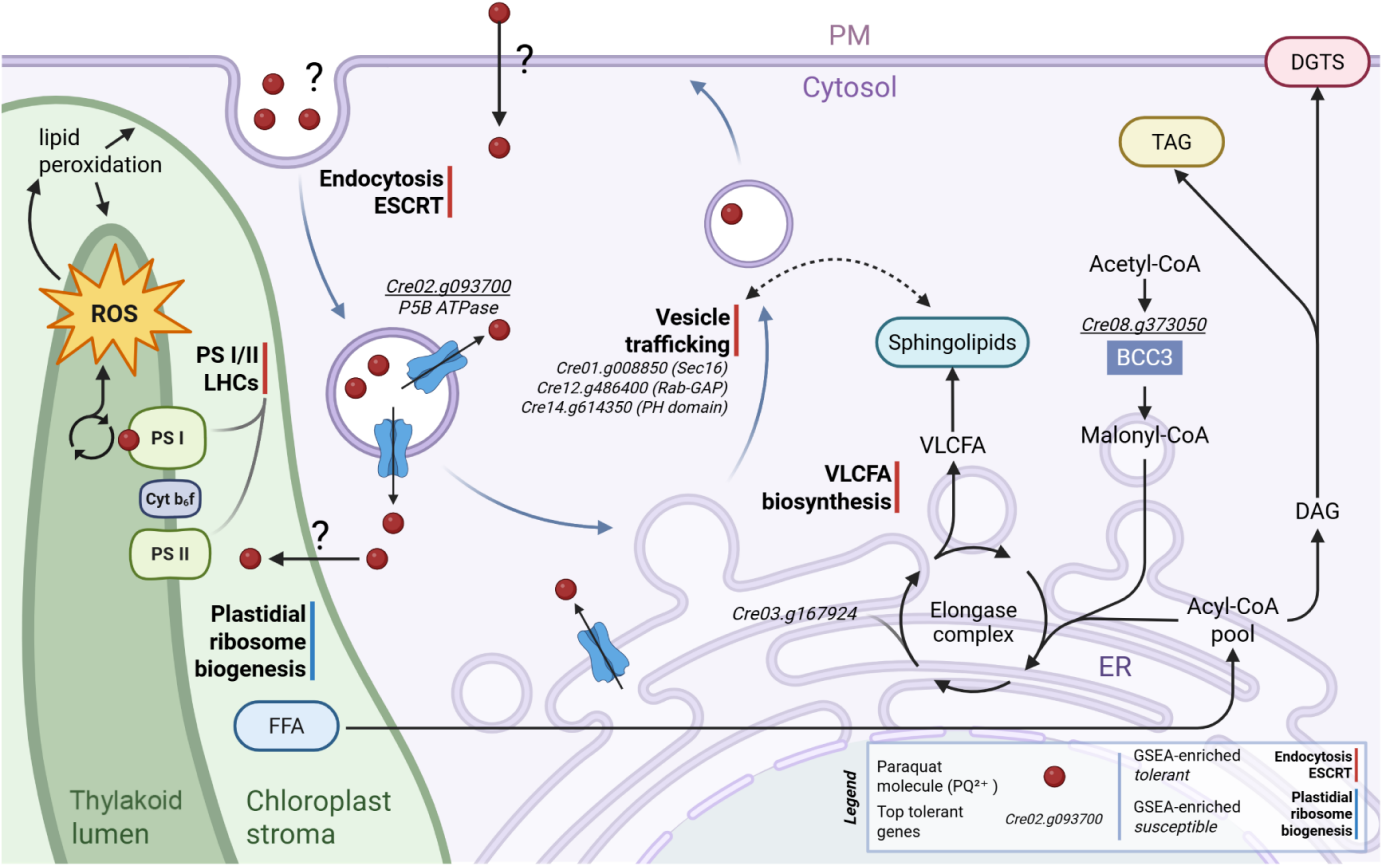
Proposed mechanisms of paraquat tolerance in *C. reinhardtii*. Paraquat (PQ^2+^, red circles) enters the cell and accumulates in endomembrane compartments. The P5B ATPase (Cre02.g093700), localized to the ER and plasma membrane, is proposed to export PQ^2+^ into the cytosol, from where it reaches PSI and generates ROS. Loss of this transporter sequesters PQ^2+^ within endomembrane compartments, preventing access to PSI. Vesicle trafficking components enriched among tolerant mutants in GSEA (Cre01.g008850/Sec16, Cre12.g486400/Rab GAP, Cre14.g614350/PH domain) may facilitate endosomal delivery of PQ^2+^. BCC3 (Cre08.g373050) provides malonyl-CoA for the ER-localized elongase complex, including Cre03.g167924 (VLCFA 3-hydroxyacyl-CoA dehydratase), to produce VLCFAs that feed into sphingolipid biosynthesis. The dashed arrow indicates the proposed but not yet experimentally confirmed link in *C. reinhardtii* between sphingolipid depletion and impaired vesicle trafficking, based on established dependencies in yeast and plants. Question marks denote steps where the molecular identity of the transporter or route is unknown. Gene IDs in italics denote top tolerant hits from Table 1; underscored gene IDs indicate the two experimentally characterized mutants. Red and blue bars next to processes indicate GSEA enrichment direction (tolerant or susceptible). PM, plasma membrane; ER, endoplasmic reticulum; PSI/PSII, Photosystem I/II; LHC, light-harvesting complex; VLCFA, very long chain fatty acid; DGTS, diacylglyceryl-N,N,N-trimethylhomoserine; TAG, triacylglycerol; DAG, diacylglycerol; FFA, free fatty acid; ROS, reactive oxygen species.

P5B ATPase provides the most direct evidence. Its ER and plasma membrane localization, structural compatibility with paraquat, and functional analogy to mammalian ATP13A2 support a role in exporting paraquat from endomembrane compartments into the cytosol, from where it reaches the PSI. The unchanged total intracellular paraquat concentration is consistent with endomembrane sequestration. That the trafficking system is required for paraquat toxicity is independently supported by three additional top hits encoding core trafficking components (Sec16, Rab GAP, PH domain protein), and by GSEA enrichment of ESCRT, CORVET, SNARE, and Rab GTPase gene sets among tolerant mutants, and by the opposing phenotypes of individual inositol phosphate pathway genes matching the known roles of specific phosphoinositide species in endomembrane compartment identity.

We propose that the VLCFA biosynthesis genes connect to this same system. VLCFAs are essential precursors for sphingolipid synthesis, and sphingolipids are, in turn, required for vesicle tethering, docking, and fusion in yeast^44^ and vesicle-mediated protein trafficking in *Arabidopsis*.^45–47^ The enrichment of ceramide synthesis genes among tolerant mutants further links VLCFA elongation, sphingolipid synthesis, and trafficking to a single functional axis. We therefore propose that VLCFA mutants confer tolerance through sphingolipid depletion, which impairs the same endomembrane delivery route disrupted by the P5B ATPase. Our lipidomics data partly (sphingolipids were below detection in both mutant and wild-type strains) support this interpretation: the BCC3 mutant shows constitutive remodeling of extraplastidial lipids, while chloroplast galactolipids remain unaffected, and its ultrastructure is completely preserved under paraquat exposure, consistent with a mechanism that prevents paraquat from reaching its target. It should be noted that endosomal trafficking and its regulation by sphingolipids remain poorly characterized in *C. reinhardtii*. While our evidence collectively builds a strong case, the specific steps by which paraquat moves from endomembrane compartments to the chloroplast and how sphingolipid composition regulates this process in algae remain to be elucidated.

This study demonstrates that genome-wide functional genomics can be successfully applied to investigate environmentally relevant chemical toxicity in a photosynthetic organism, yielding mechanistic resolution that conventional ecotoxicological approaches cannot provide. Although the primary targets of both paraquat and diuron are chloroplast-encoded, our screen mapped the critical nuclear architecture of nontarget-site resistance and established direct links between specific genes and fitness under herbicide stress. For paraquat, this included experimentally validated nontarget-site resistance mechanisms involving intracellular trafficking and lipid metabolism. For diuron, the identified candidate genes point to a plausible NADPH conservation strategy, but this interpretation remains a hypothesis pending experimental validation.

## Supplementary Material













## Notes

Supplementary table 5: Primers used for validation of insertion in mutants LMJ.RY0402.155567 and LMJ.RY0402.062151 (XLSX)
Supplemental table 6.5: Gene set enrichment analysis results of Flagellar and Greencut2 protein sets (Paraquat treatment) (XLSX)
Supplemental table 7.5: Gene set enrichment analysis results of Flagellar and Greencut2 protein sets (Diuron treatment) (XLSX)
Supplementary table 8: Raw growth data for Chlamydomonas reinhardtii wild-type (WT) and mutant strains (LMJ.RY0402.155567, Cre02.g093700; LMJ.RY0402.062151, Cre08.g373050) (XLSX)
Supplementary table 4: High-confidence gene–phenotype relationships of diuron treatment (XLSX)
(Supplementary Figures 1–8) showing replicate consistency; mutant responses to paraquat and diuron; inositol phosphate metabolism KEGG pathway; structural modeling, and lipidomic and DNA fragmentation assays; detailed experimental procedures for laddering, microscopy, LC–MS/MS, lipidomics, and statistical and structural modeling analyses; and (Supplementary Tables 1–10) including raw mutant counts, gene-phenotype associations; enrichment analyses; and docking results Supplementary Tables 1–8 provided as separate Excel files (PDF)
