# Supplementary material for: Functional Genomics Screening in Chlamydomonas reinhardtii Maps the Genetic Landscape of Tolerance to Paraquat and Diuron
Source: Environ Sci Technol. 2026 May 27;60(22):15570–85. doi: 10.1021/acs.est.5c17308 (PMC13262031; doi:10.1021/acs.est.5c17308)
Supplement: Supplementary file 6 [file es5c17308_si_006.pdf]

SUPPORTING INFORMATION

Functional Genomics Screening in Chlamydomonas reinhardtii Maps the Genetic Landscape of Tolerance to Paraquat and Diuron

Tim Godec[1,9], Carissa Bleker[1], Katja Stare[1], Tjaša Lukan[1], Valentina Levak[1,9], Magda Tušek Žnidarič[1], Alexander Betz[10], Tina Kosjek[2], Katarina P van Midden[3], Marina Klemenčič[3], Francesco Trenti[4], Graziano Guella[4], Kristina Sepčić[5], Friedrich Fauser[6], Weronika Patena[6], Martin C. Jonikas[6,7], Maruša Kerenčič[8], Tina Eleršek[8], Mélanie Pietri[13,14], Thomas Rodet[14], Urban Bren[15,16,17], Marko Jukić[15,16], Samo Lešnik[15,17], Anže Županič[1,10,11]*

[1] National Institute of Biology, Department of Biotechnology and Systems Biology, 1000 Ljubljana, Slovenia
[2] Jožef Stefan Institute, Department of Environmental Sciences, 1000 Ljubljana, Slovenia
[3] University of Ljubljana, Faculty of Chemistry and Chemical Technology, 1000 Ljubljana, Slovenia
[4] University of Trento, Department of Physics, 38123 Povo, Trento, Italy
[5] University of Ljubljana, Biotechnical Faculty, 1000 Ljubljana, Slovenia
[6] Princeton University, Department of Molecular Biology, Princeton, New Jersey 08544, United States
[7] Howard Hughes Medical Institute, Princeton, New Jersey 08544, United States
[8] National Institute of Biology, Department of Genetic Toxicology and Cancer Biology, 1000 Ljubljana, Slovenia
[9] Jožef Stefan International Postgraduate School, Ljubljana, Slovenia
[10] Eawag - Swiss federal institute of aquatic science and technology, 8600 Duebendorf, Switzerland
[11] University of Ljubljana, Faculty of Computer and Information Science, 1000 Ljubljana, Slovenia
[12] University Paris-Saclay, ENS Paris Saclay, CNRS, LuMIn, 91190 Gif-sur-Yvette, France
[13] University Paris-Saclay, CNRS, ENS Paris Saclay, LMF, 91190 Gif-sur-Yvette, France
[14] University Paris-Saclay, ENS Paris Saclay, CNRS, Satie, 91190 Gif-sur-Yvette, France
[15] University of Maribor, Faculty of Chemistry and Chemical Engineering, Laboratory of physical chemistry and chemical thermodynamics, 6000 Maribor, Slovenia
[16] Faculty of Mathematics, Natural Sciences and Information Technologies, University of Primorska, 6000 Koper, Slovenia
[17] Institute of Environmental Protection and Sensors, 2000 Maribor, Slovenia
*Email: anze.zupanic@nib.si

LIST OF SUPPORTING INFORMATION

Number of pages: 23

Number of Supplementary figures: 8

Number of Supplementary tables: 10

SUPPLEMENTARY FIGURES

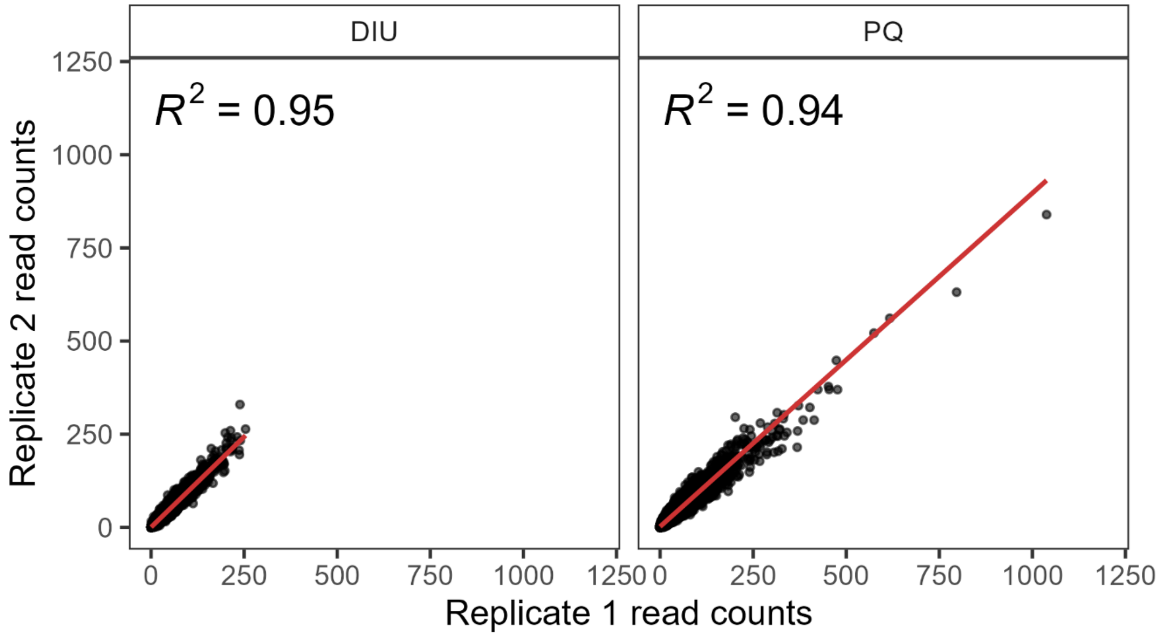

Supplementary Figure 1. Correlation of barcode read counts between biological replicates for diuron and paraquat treatments (R^2 = 0.95 and R^2 = 0.94, respectively). DIU: diuron exposure, PQ: paraquat exposure.

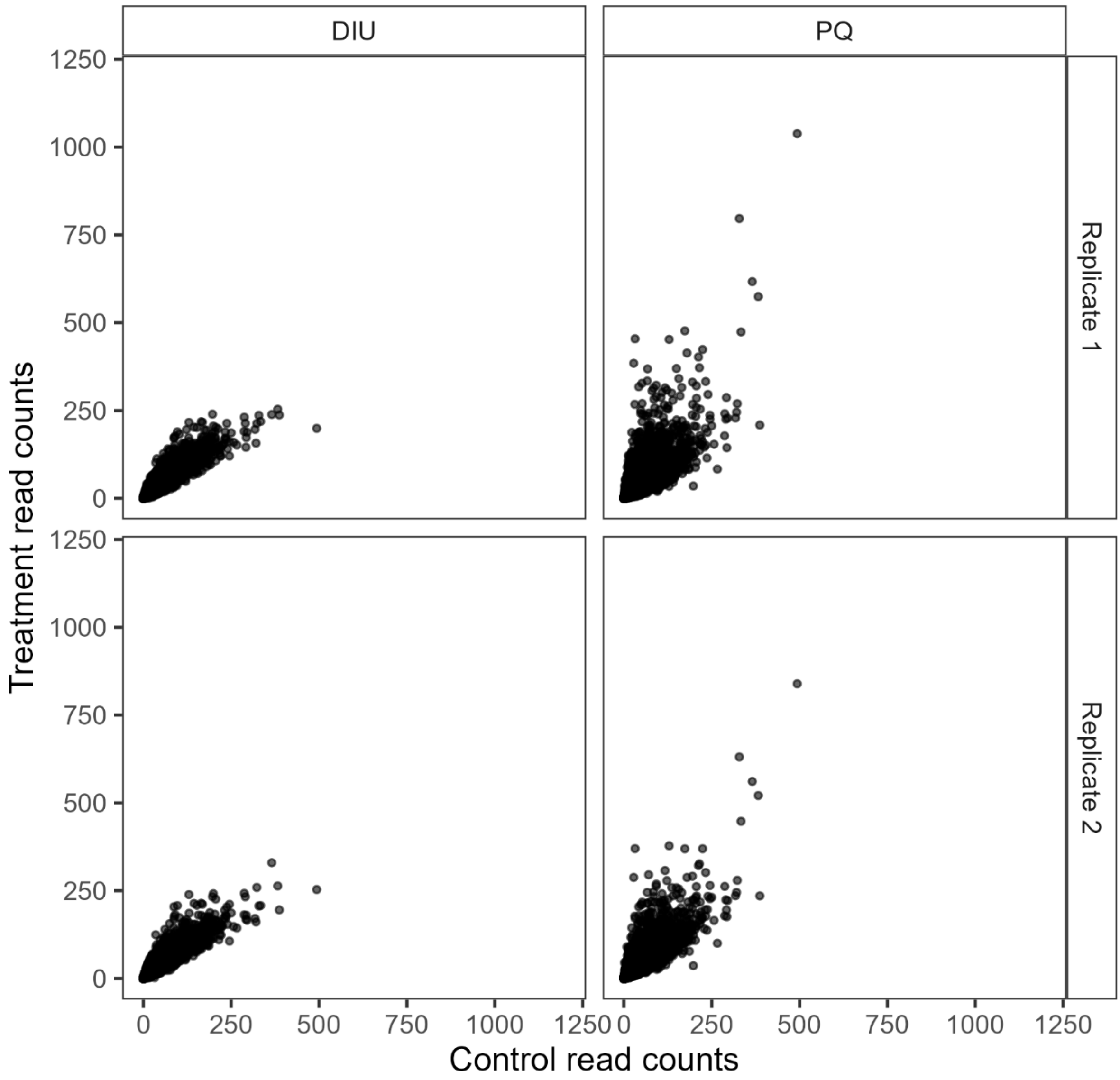

Supplementary Figure 2. Comparison of mutant read counts between control and treatment samples for each replicate. DIU: diuron exposure, PQ: paraquat exposure.

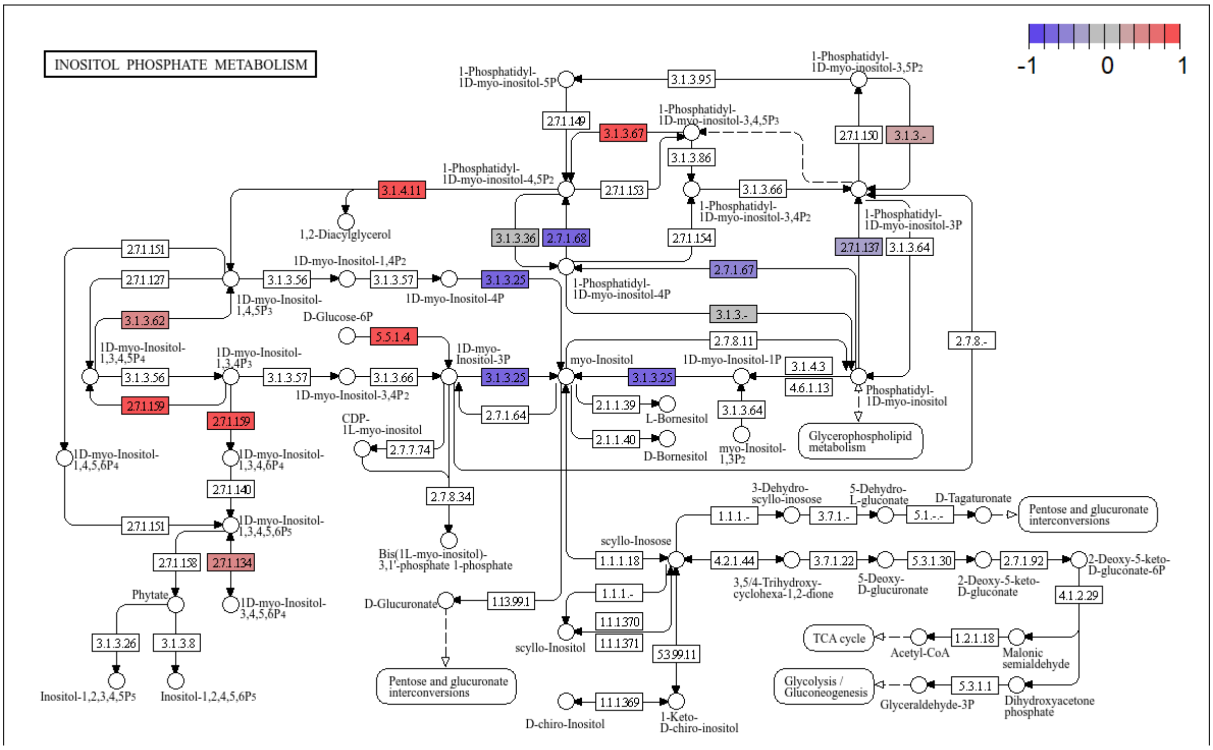

Supplementary Figure 3. Inositol phosphate metabolism KEGG pathway (cre00562) colored by mutant phenotype under paraquat treatment. Enzyme nodes are colored according to the growth phenotype of their corresponding gene disruption: red indicates tolerance (positive $\log_2$ fold change), blue indicates susceptibility (negative $\log_2$ fold change), gray indicates near-zero $\log_2$ fold change, and white indicates genes without orthologs in *C. reinhardtii* or without mutant coverage in the library. Enzymes producing endosome-associated PtdIns(3)P and soluble inositol phosphates (IP_3, IP_4, IP_5) are associated with tolerance upon disruption, while those required for myo-inositol and core phosphatidylinositol biosynthesis are associated with susceptibility. Pathway rendered using Pathview with *C. reinhardtii* KEGG annotations.

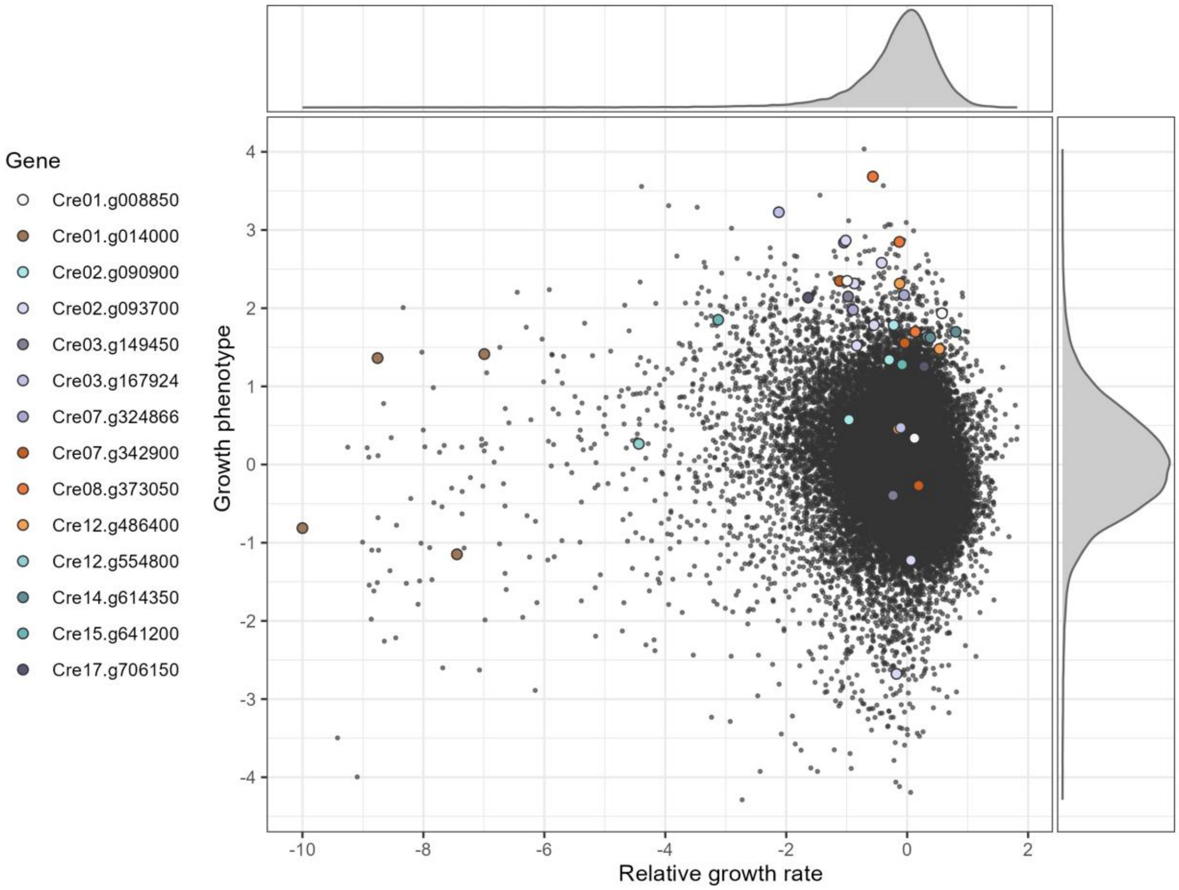

Supplementary Figure 4. Relationship between baseline growth rate and paraquat tolerance phenotype. Scatterplot of paraquat growth phenotype (log_{10} fold change, y-axis) versus relative growth rate under standard conditions (x-axis data from Fauser et al., 2022, licensed under CC BY 4.0) for all mutants retained in both datasets. Grey dots represent individual mutants; colored dots highlight the top paraquat-tolerant hits from Table 1, with gene identities in the legend. Marginal density plots show the distribution along each axis. While the top tolerant mutants tend toward slower baseline growth, the majority of slow-growing mutants show no paraquat tolerance, consistent with gene-specific rather than growth rate-dependent mechanisms.

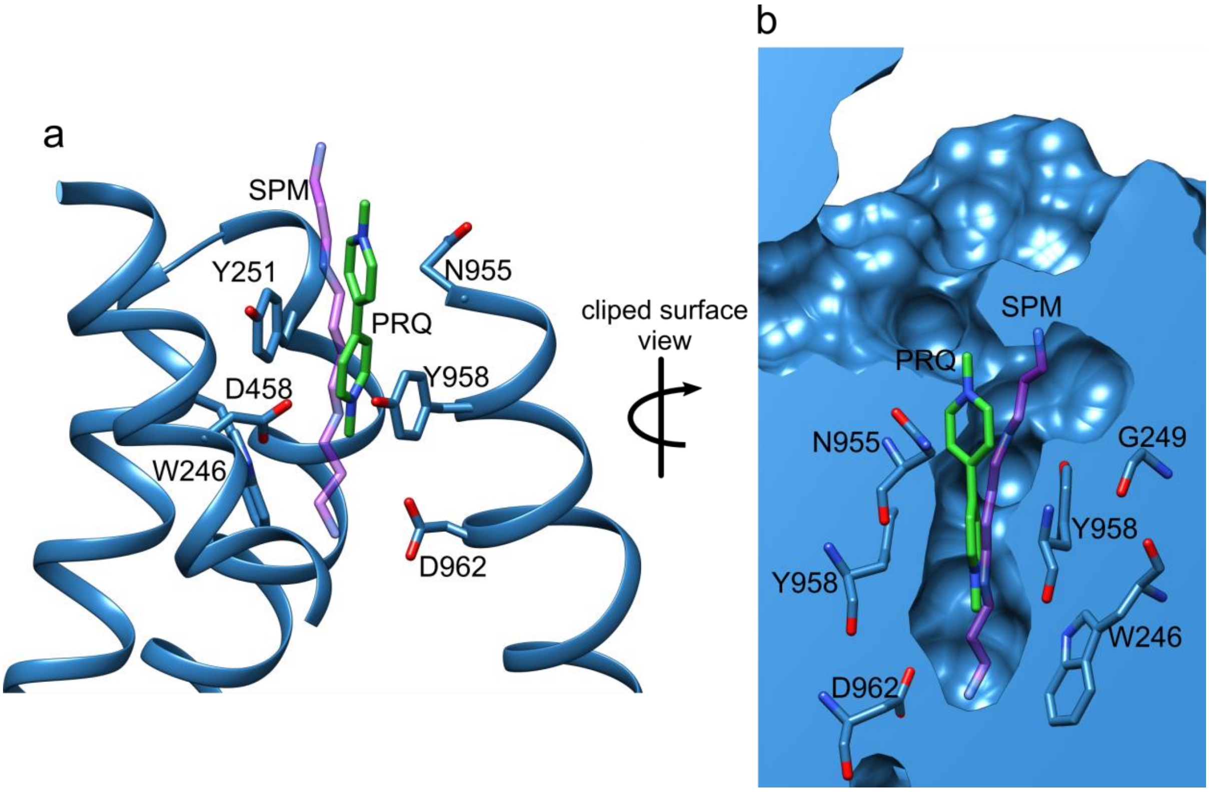

Supplementary Figure 5. Docking of paraquat (PRQ) into the *ATP13A2-like* polyamine-binding pocket. (a) Binding-site α-helices with the docked PRQ pose (green). SPM (purple) is included only as an orientation reference, positioned by superposition to the template structure (PDB ID: 7N72). (b) Clipped and rotated surface view of the same pocket highlighting the elongated cavity in the E2-like model.

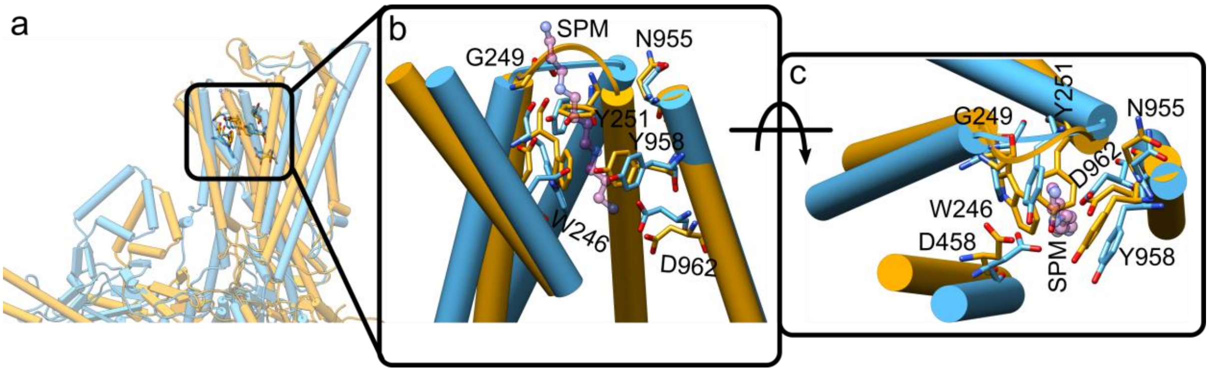

Supplementary Figure 6. Conformational comparison of the *ATP13A2-like* polyamine-binding site (model 1 vs model 6; see Supplementary table 9). (a) Superposition of the E1-apo conformation (model 1, orange) and the E2–Pi-like conformation (model 6, blue). (b) Close-up view of the binding-site α-helices showing the residues lining the pocket. For orientation, spermine (SPM) was positioned by superposing model 6 onto its template structure (PDB ID: 7N72) and is displayed as a transparent ball-and-stick representation. (c) Same region as in (b), rotated to highlight the approximately cylindrical pocket geometry and the rearrangement of helices between the E1 and E2–Pi-like conformations.

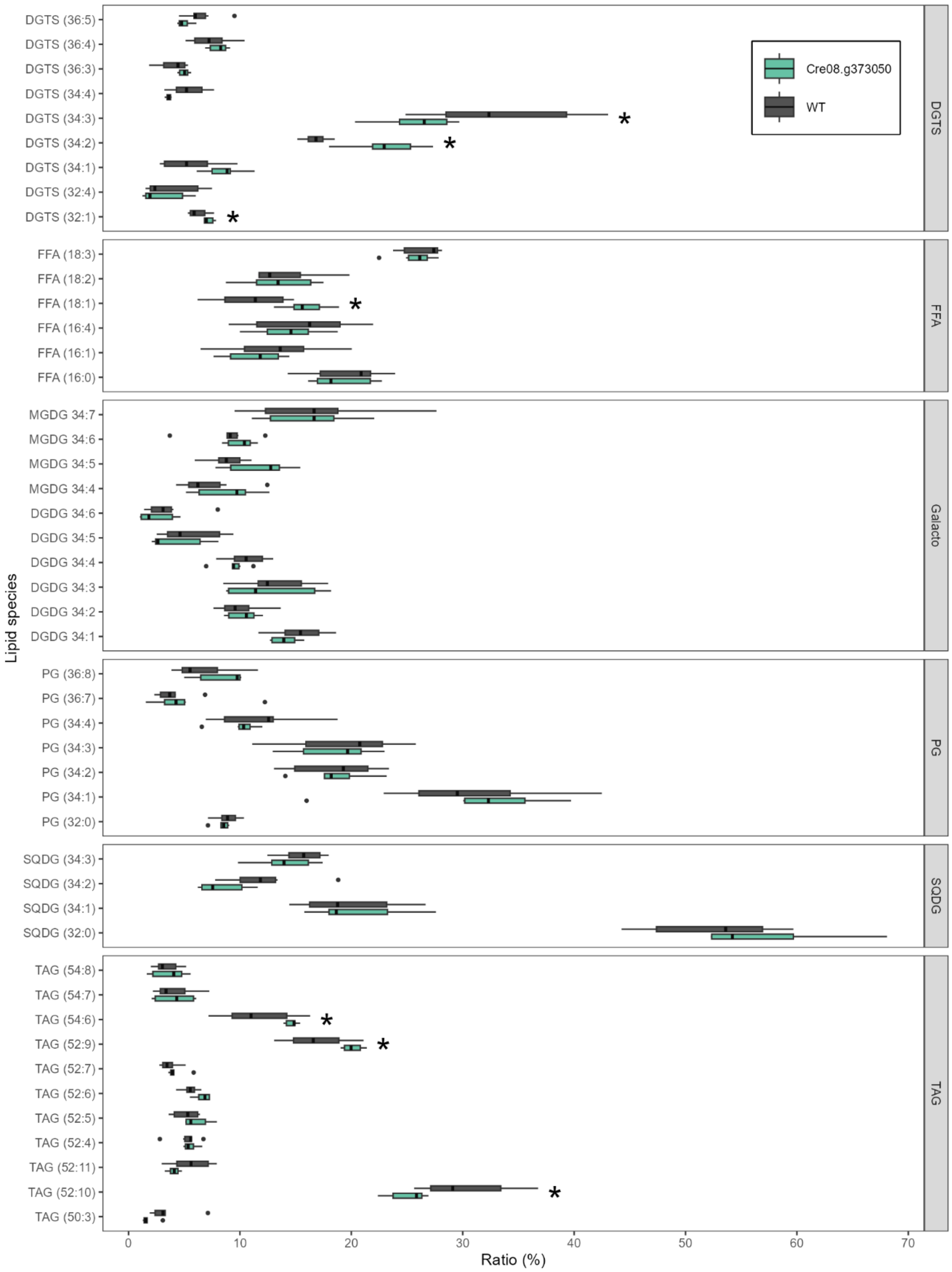

Supplementary Figure 7. Lipid species ratios of lipid groups for wilde-type (WT) and Cre08.g373050 mutant. Species with ratio <5% were omitted. Lipid species with significant differences (FDR-adjusted $p < 0.1$) are labelled with "*". DGTS: diacylglyceryltrimethylhomoserines, FFA: free fatty acids, Galacto: galactolipids, PG: phosphatidylglycerols, SQDG: sulfoquinovosyl diacylglycerols, TAG: triacylglycerols

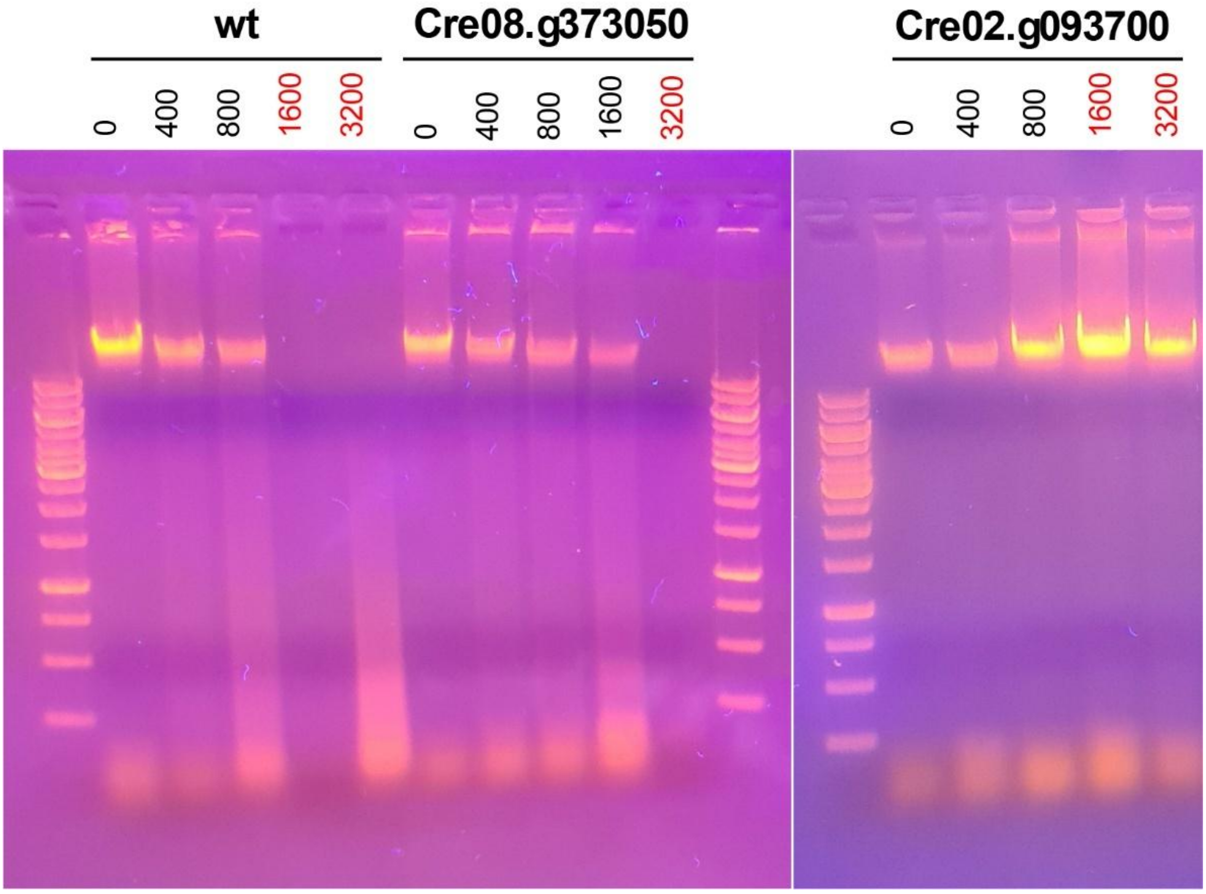

Supplementary Figure 8. DNA laddering assay of Chlamydomonas reinhardtii wild type (WT) and mutant strains Cre02.g093700 (putative transporter) and Cre08.g373050 (acetyl-CoA carboxylase). Genomic DNA was separated on an agarose gel to assess fragmentation patterns indicative of apoptosis. Paraquat concentrations (nM) are indicated above each sample; concentrations marked in red represent those with insufficient DNA yield to load the target amount of 6 µg, and all available DNA was therefore used. No DNA laddering pattern was observed in any of the tested strains, indicating absence of apoptotic DNA fragmentation.

SUPPLEMENTARY METHODS

DNA extraction and laddering

Cells were thawed on ice and 250 µL DNA extraction buffer (50 mM Tris-HCl pH = 8.0, 20 mM EDTA, 200 mM NaCl, 1% SDS) was added. DNA extraction was performed using the phenol/chloroform/isoamyl alcohol mixture (25:24:1, Sigma Aldrich, cat. nr 77617). Briefly, 400 uL of the mixture was added to each sample, vortexed for 1-2 min and centrifuged at 21.000 × g for 5 min, and the aqueous solution was collected in a fresh tube. This was repeated once more. Next, 2 µL RNAse A (Thermo Scientific, EN0531) was added to each sample and incubated at room temperature for 1 h. After incubation, 400 uL of the phenol/chloroform/isoamyl alcohol mixture was added, vortexed and centrifuged as before. A final extraction with 400 uL chloroform/isoamyl alcohol (24:1 ratio) was performed and the aqueous fraction collected. Genomic DNA was precipitated by the addition of 1 ml of absolute ethanol, mixed by inverting the tube and incubated overnight at -20 °C. Next day the genomic DNA was pelleted by centrifugation at 21.000 × g for 20 min. The pellet was washed once with 70% ethanol and dissolved in 30 uL of nuclease free water. The concentrations were measured and if the amount of DNA was lower than 6 µg, all extracted DNA was loaded onto the gel.

Confocal microscopy - acquisition parameters

To study Cre02.g093700 (GFP-labelled) colocalization with nucleus (RFP-labelled) or plasma membrane (mCherry-labelled) or chloroplasts (chlorophyll fluorescence), the florescence was visualized with confocal microscope Leica Stellaris 8. The 488 nm laser was used for excitation of GFP and the white laser for excitation of RFP, mCherry or chlorophyll fluorescence. The GFP, RFP, mCherry and chlorophyll emissions were measured in the windows 505–525 nm, 585–630 nm, 605–630 nm, and 690–750 nm, respectively and were collected simultaneously through three channels using a 20x objective. To study Cre02.g093700 and ER colocalization with confocal microscope Leica Stellaris 5, the 488 nm laser was used for the excitation of GFP and the 561 nm laser for excitation of RFP. The GFP and RFP emissions were measured in the windows 500–550 nm and 605–670 nm, respectively, and were sequentially collected through two channels using a 20x objective. Regions of interest (ROI) were bidirectionally scanned within agroinfiltrated area with a resolution of 1024 x 1024 pixels, frame average 2 and scan speed 400 Hz.

Intracellular paraquat measurements - LC-MS/MS analysis

Paraquat was extracted from the cell lysates with one mL of extraction solvent methanol/water (8/2 vol./vol.) with 0.1% formic acid followed by an addition of 10 µL of 10 µg/mL isotope labeled internal standard (Paraquat d8, purity, Dr. Ehrenstorfer). This mixture was then vortex mixed for two minutes, incubated at 80 °C for 15 min and centrifuged at 4000 rpm for 15 minutes. The clear supernatant was transferred to vials and analyzed using a Nexera ultra high-performance LC (Shimadzu Corp., Kyoto, Japan) coupled with a QTRAP® 4500 MS/MS system (AB Sciex, Darmstadt, Germany). The liquid chromatograph was equipped with 5-cm Raptor HILIC-Si column with a particle size of 2.7 µm and an inner diameter of 2.1 mm. The mobile phases were (B) Water with 50 mM ammonium formate and 0.5% formic acid, and (A) water/acetonitrile (25/75, vol./vol.) with 50 mM ammonium formate and 0.5% formic acid. The flow rate was 0.6 mL/min and 5 µL volume was injected. The gradient started at 100% A and was decreased to 65% A within 4 minutes, then increased back to 100% A in 0.1 min and left so for 3 minutes to allow the column to equilibrate. The mass spectrometer was operated under positive electrospray ionization in multiple reaction monitoring acquisition mode, where one quantitative (185>170) and one qualitative (171>77) transitions were followed for paraquat eluting at the retention time of 3 min. The MRM transitions for the internal standard were 193>178 and 194>178, the first one being used for normalization. The instrument was operated by Analyst v1.6.3, which was also used for raw MS data processing.

The method validation parameters involved determining the linearity range of the matrix-matched calibration curve between 2.5 ng/mL and 1 µg/mL, with a linear regression quotient 0.998. The quality of sample preparation and analysis was monitored through solvent blanks, process blanks and quality control samples. The latter were prepared by spiking the blank pellet suspension with the analyte and internal standard at three concentration levels (LOQ, medium and high). The accuracy was within ± 23% and the precision was better than ± 12%.

Lipidomics - chromatographic and mass spectrometry parameters

Each pellet was resuspended in 1 mL of 0,9% NaCl, then supplemented with 3.75 mM chloroform:methanol (2:1, v:v), and vigorously vortexed for 15 min. The procedure was followed by a sequential addition of 1.25 mL chloroform and 1.25 mL deionized water, each of these steps followed by a 1-minute vortexing. The samples were centrifuged for 5 minutes at 1050 × g, the upper aqueous phase was carefully discharged, and the lower phase (total lipid extract) was used for further lipidomic analyses.

Total lipid extracts were analyzed by liquid chromatography–mass spectrometry (LC-MS) (Model 1100 series; Hewlett- Packard) coupled to a quadrupole ion-trap mass spectrometer (Esquire LCTM; Bruker, Bremen, Germany).

Chromatographic separation of the phospholipids was carried out at 303 K on a C18 column (Kinetex TMC18; length, 100 mm; particle size, 2.6 μm; internal diameter, 2.1 mm; pore size, 100 Å; Phenomenex, Torrence, CA, USA). The solvent system consisted of eluant A as MeOH/H2O (7:3, v/v) containing 10 mM ammonium acetate and eluant B as isopropanol/MeOH (10:90, v/v) containing 10 mM ammonium acetate. Samples were resuspended in 1 mL CHCl3/MeOH (2:1, v/v), and 10 μL was run with a linear gradient from 65% eluant B to 100% B in 40 min, plus 20 min isocratic 100% B at 1 mL/min. The column was then requilibrated to 65% B for 10 min.

The MS scan range was 13,000 U/s in the range of 50 to 1500 m/z, with a mass accuracy of ~100 ppm. The nebulizer gas was high purity nitrogen at a pressure of 20 to 30 psi, at a flow rate of 6 L/min and at 300 °C. The electrospray ionization was operated in positive and negative ion modes. For the structural assignments of the lipid species, the extracted ion chromatograms from the positive and/or negative ion full scan data were integrated using the DataAnalysis 3.0 software (Bruker Daltonik, Bremen, Germany).

Alternative statistical approach using Gaussian Mixture Model and Bayesian estimators

To complement Fisher's exact test, which can be sensitive to outliers when sample sizes are small, we developed a two-step probabilistic approach to identify genes conferring herbicide (Paraquat or Diuron) sensitivity or tolerance.

1) *Mutation-level detection*

For each mutation, we considered two hypotheses:

- $\mathcal{H}_0$: the mutation does not affect sensitivity or tolerance to the herbicide
- $\mathcal{H}_1$: the mutation affects sensitivity or tolerance to the herbicide

We modeled the read count in treatment condition for each mutation using a Gaussian mixture model (GMM) with two components, allowing estimation of the probability that a mutant responds to the treatment. Detection probability p_d was computed for each mutant based on the relative likelihood of the data under $\mathcal{H}_0$ versus $\mathcal{H}_1$. Mutations with a detection probability below 0.5 were considered likely to show a significant response.

2) *Gene-level aggregation*

For each gene, let n denote the number of mutants, accounting for replicates. Mutant-level probabilities were combined using two Bayesian estimators:

- *Maximum A Posteriori (MAP).* $\hat{p}_{MAP} = \sum_{i=1}^{n} p_d^i / n$ the most likely detection probability given the observed data,
- *Expectation A Posteriori (EAP).* $\hat{p}_{EAP} = (\sum_{i=1}^{n} p_d^i + 1)/(n+2)$ a regularized estimate that incorporates prior uncertainty, particularly useful when the number mutants per gene is low.

The variance of the EAP estimator is:

$$\mathbb{V}[\hat{p}_{EAP}] = \frac{(1 + \sum_{i=1}^{n} p_d^i)(n + 1 - \sum_{i=1}^{n} p_d^i)}{(n+2)^2(n+3)}$$

Each mutation can be considered as a Bernoulli trial that is either detected (sensitive or tolerant) or not. Under this assumption, the total number of detected mutants per gene follows a binomial distribution. Assuming a uniform prior on [0,1] for p_d, the posterior distribution of p_d becomes a Beta distribution. The MAP corresponds to the observed

fraction of “successes,” while the EAP provides a Bayesian estimate of the success probability, effectively combining the binomial framework with a uniform prior on [0,1]. The EAP estimator also allows computation of the variance of the detection probability at the gene level, providing a measure of confidence not available from Fisher’s exact test alone.

This two-step approach - mutant-level detection using a GMM and gene-level aggregation using Bayesian estimators - offers a robust complement to traditional Fisher’s exact test, particularly in datasets with few replicates or potential outliers.

In silico structural analysis and homology modelling

We set an *in silico* modeling campaign to support the tructural analysis of identified Cre02.g093700 from *Chlamydomonas reinhardtii* (https://phytozome-next.jgi.doe.gov/report/gene/Creinhardtii_v5_6/Cre02.g093700). First, corresponding UniProt A0A2K3E1F7 (A0A2K3E1F5) protein sequences were checked for secondary structure and domain identification using InterPro to immediately identify a P-type ATPase, subfamily V (identified domains: P5-type ATPase cation transporter – IPR047819; IPR023298, IPR059000, IPR023299, IPR023214) supporting the Phytozome auto notation (cation-transporting ATPase 13A3/4/5). Phobius topology prediction (https://phobius.sbc.su.se/) indicated a multi-pass membrane protein comprising eleven transmembrane helices (residues 71–88, 264–281, 287–305, 456–480, 492–513, 1177–1199, 1205–1224, 1245–1267, 1291–1309, 1321–1340, and 1370–1392), whereas TMHMM predicted nine core helices with two additional segments falling near the confidence threshold.

The three-dimensional structure model of A0A2K3E1F7 was then constructed using homology modeling. Template identification was performed by three iterative PSI-BLAST searches against UniRef90 to construct a position-specific scoring matrix (PSSM), which was subsequently used to query the Protein Data Bank (PDB). Templates with E-values ≤ 0.5 were considered, resulting in 129 significant structural hits. Homology campaign was promising with top hits (PDB ID: 7N75, 7N70) being a polyamine transporter (Organism: *Homo sapiens*) ATP13A2 from P5B ATPase family (DOI: 10.1016/j.molcel.2021.08.017). Top hits demonstrated high coverage, alignment scores > 1100 and the templates corresponded to experimentally determined Cryo-EM structures with resolutions 2.8–3.7 Å. Top hit (PDB ID: 7N75) template aligned 953 from 1439 (66.2%) residues with 38.0% sequence identity and 57.1% sequence similarity (*Supplementary information*: sequence alignment).

Homology modeling was performed using hm_build protocol (version 25.12.1) (DOI: doi.org/10.1007/978-1-4939-7366-8_4). Parameters were five alignment variants per template (Alignments = 5), loop regions sampled with up to 50 conformations per loop (LoopSamples = 50), and terminal extensions were limited to 10 residues to avoid miss-interpretation of poorly supported regions. Models were constructed in the monomeric state (OligoStateMin = 1; OligoStateMax = 4). Side-chain conformations were optimized using dead-end elimination, followed by full-atom energy refinement under the YASARA force field with an explicit solvent to improve the model stereochemical quality (DOI: 10.1002/prot.10104).

Structural robustness was evaluated using per-residue and global quality Z-scores, including dihedral, packing 1D, and packing 3D metrics. The top-ranked model, built as a monomer from template 7N75-A (alignment variant 03), achieved an overall Z-score of −1.280 for residues 59–1423. Backbone stereochemistry was classified as optimal, with

a dihedral Z-score of 0.562, indicating geometry comparable to high-resolution experimental structures. Side-chain packing quality was within acceptable homology-model ranges, with packing 1D and packing 3D Z-scores of −1.951 and −1.291, respectively. Moreover, all five highest-ranking models were all derived from the same ATP13A2 template (7N75-A) and exhibited closely clustered overall Z-scores (−1.280 to −1.330). This indicates strong convergence with respect to both template selection and alignment uncertainty. Collectively, the homology modeling results demonstrate robust convergence toward a P-type ATPase architecture consistent with the ATP13A2 structural family.

To further examine the ATP13A2 postulation, we employed AlphaFold3 (AF) (DOI: 10.1038/s41586-024-07487-w) to model our identified Cre02.g093700 only to obtain models with an average pTM = 0.7 that closely align the central TM region to our homology model with an overall RMSD of 3.5 Å (mostly due to AF differences primarily observed in low-confidence regions outside of the transmembrane core). Moreover, model comparison using Dali platform (http://ekhidna2.biocenter.helsinki.fi/dali/) suggested a close ATP13A2 human polyamine transporter homology with Dali top hits being PDB ID: 7n78, 7fjm, 7vpk, 7n72, 7vpj, 7n76, 7fjq, 7n74 with average 36 % identity (DOI: 10.1038/s41467-023-37741-0). A0A2K3E1F7 FASTA sequence also reveals the presence of the conserved PP(A/V)L motif, which is characteristic of the P5B-type ATPase subfamily (DOI: 10.1016/j.bbamcr.2022.119354, 10.1016/j.neuint.2012.01.002, 10.1038/s41421-021-00334-6, 0.1038/s41467-023-37741-0). Collectively, sequence, structure, and topology-based analyses support classification of A0A2K3E1F7 as a P5B-type ATPase.

Molecular docking and transporter state homology modeling

To elaborate on the functional role of our structure - homology model, we further modeled all related conformational states described for the ATP13A2 (sister 7N75 top templates) by Sue Im Sim et al. (DOI: 10.1016/j.molcel.2021.08.017), including the E2P-like (PDB ID 7N70), E2-Pi–like (PDB ID 7N72), E2-Pi post-hydrolysis (PDB ID 7N78), E1P–ADP–like (PDB ID 7N73), E1–ATP (PDB ID 7N74), E1–apo (PDB ID 7N75 and 7N76), and E1P-like (PDB ID 7N77) states. Independent homology models were generated for each conformer using identical homology modeling parameters as described above (all models were satisfactory with Z-scores ranging from -1.252 to -1.687). The resulting models reproduced the characteristic large-scale movements of the A-, N-, P-domains and the coordinated movements of transmembrane helices that define the E1–E2 conformational transition. Observations are consistent with the dynamic transport mechanism characteristic of P5B-type polyamine transporters, and the examined protein could function as polyamine/paraquat transporter as long as the binding site itself is functionally intact.

Therefore, binding site function was inspected by superposition and state-dependent molecular docking to test our hypothesis that if our candidate protein is an ATP13A2/P5B-like transporter, then substrate accessibility should be strongly conformation-dependent, with the luminal cavity able to bind paroquat predominantly in E2-family states and be comparatively occluded in E1/E1P states. Molecular docking of paraquat (PRQ) as a probe was therefore used as a binding site integrity study across the transport cycle. The goal was not to infer exact binding affinities, but to assess whether the studied PRQ and spermine (SPM, crystal ligand) can be accommodated in the expected luminal site only in the expected conformations.

State-dependent docking was performed across our ATP13A2 conformational ensemble, using homology models built for eight representative P-type ATPase cycle states based on the available ATP13A2/P5B-ATPase cryo-EM templates (PDB IDs 7N70, 7N72, 7N73, 7N74, 7N75, 7N76, 7N77, 7N78). Using an identical Vinardo/AutoDock Vina setup (DOI: 10.1371/journal.pone.0155183) for all states (grid centered on the putative luminal substrate cavity; 18 × 18 × 18 Å; exhaustiveness 128), we docked SPM and PRQ into each model. In these molecular dockings, both ligands were treated in their fully charged (physiologically relevant) forms: spermine as a fully protonated polycation at physiological pH, and paraquat as a permanent dication. Across all E1-like conformations (E1_apo_7N75, E1_apo_7N76, E1_ATP_7N74) and E1P-like conformations (E1P_ADPlike_7N73, E1P_like_7N77), the top-ranked poses for both ligands failed to occupy the expected substrate cavity. In contrast, in the E2-family conformations (E2_Pi_like_7N72, E2_Pi_post_hydrolysis_7N78, and E2P_like_7N70), both SPM and PRQ consistently docked into the binding-site region, which is the luminal cavity lined by residues previously implicated in the polyamine site (including W246, G249, Y251, D458, N955, Y958, and D962 (Supplementary table 9, Supplementary Figure 6). Vina-family scoring functions (including Vinardo) provide approximate binding scores and only partially capture protein flexibility, explicit solvent/water mediation, and entropic effects; limitations that are particularly relevant for highly charged ligands such as polyamines and paraquat. However, herein, we used molecular docking to primarily support the state dependence and demonstrated binding site integrity and expected pose plausibility with a consistent relative preference for E2 conformations (E1/E1P vs E2 accessibility) (Supplementary Figure 7).

SUPPLEMENTARY TABLES

Supplementary tables 1-8 are available as separate (xlsx) files.

Supplementary table 1:
Mutant raw and normalised counts with metadata

Supplementary table 2:
Mutant phenotypes of alleles per treatment and replicate

Supplementary table 3:
High-confidence gene–phenotype relationships of paraquat treatment

Supplementary table 4:
High-confidence gene–phenotype relationships of diuron treatment

Supplementary table 5:
Primers used for validation of insertion in mutants LMJ.RY0402.155567 and LMJ.RY0402.062151

Supplementary table 6:
Gene set enrichment analysis results (Paraquat treatment)

Supplementary table 7:
Gene set enrichment analysis results (Diuron treatment)

Supplementary table 8:
Raw growth data for Chlamydomonas reinhardtii wild-type (WT) and mutant strains (LMJ.RY0402.155567, Cre02.g093700; LMJ.RY0402.062151, Cre08.g373050)

Supplementary table 9. Qualitative state dependence of spermine (SPM) and paraquat (PRQ) docking into the putative luminal substrate cavity of ATP13A2-like models.

Model number	Model	SPM docks in substrate cavity?	PRQ docks in substrate cavity?
1	E1_apo_7N75_t001_a02	No	No
2	E1_apo_7N76_t001_a05	No	No
3	E1_ATP_7N74_t001_a03	No	No
4	E1P_ADPlike_7N73_t001_a05	No	No
5	E1P_like_7N77_t001_a03	No	No
6	E2_Pi_like_7N72_t001_a05	Yes	Yes
7	E2_Pi_post_hydrolysis_7N78_t001_a03	Yes	Yes
8	E2P_like_7N70_t001_a03	Yes	Yes

Supplementary table 10. Best docking scores of PRM and SPM ligands docked into the E1-like and E2-like models.

Model	Ligand	Best docking score
E1_apo_7N75_t001_a02	PRQ	-2.4
E1_apo_7N75_t001_a02	SPM	-3.3
E1_apo_7N76_t001_a05	PRQ	-1.9
E1_apo_7N76_t001_a05	SPM	/
E1_ATP_7N74_t001_a03	PRQ	-2.9
E1_ATP_7N74_t001_a03	SPM	-3.0
E1P_ADPlike_7N73_t001_a05	PRQ	-2.4
E1P_ADPlike_7N73_t001_a05	SPM	-2.9
E1P_like_7N77_t001_a03	PRQ	-3.5
E1P_like_7N77_t001_a03	SPM	-3.7
E2_Pi_like_7N72_t001_a05	PRQ	-3.4
E2_Pi_like_7N72_t001_a05	SPM	-4.6
E2_Pi_post_hydrolysis_7N78_t001_a03	PRQ	-5.4
E2_Pi_post_hydrolysis_7N78_t001_a03	SPM	-5.2
E2P_like_7N70_t001_a03	PRQ	-3.9
E2P_like_7N70_t001_a03	SPM	-4.0

OTHER SUPPORTING INFORMATION

Homology modeling alignment

SecStr :
CCCCCCCCCCCCCCCCCCCCCCCCCCCCCCCCCCCCCCCCCCCCCCCCCCCCCC
CCEEEEEEEECCCHHHHHHHHHHHHHHHHHHHHHHCCHHHHEEEEECCCCCCCCE
EEEEECCCCEEEEEEEEEECCCCCCCCCCCCCCCCCCCCCCCCHHHCCCCEEEEE
EEEEEEEECCHHHHHHHHCCCCCCCCCCCCCCCCCCCCCHHHHHHHHHHHHHC
CCCCCHHHHHHHHHHHHHHHHCCCCCCCCCCCHHHHHHHHHHCHHHHHHHHHH
HHHHHHHHHHHHHHHHHHHHHHHHHHHHHHHHHHHHHHHHHHCCCCEEEEEEECC
EEEEEECCCCCCCCEEEECCCCCCCCEEEEECCEEECCCCCCCCCCCCCCCCCCCCC
CCCCCCCCCCCCCEEECCCEEEEEECCCCCCCEEEEEECCCCCCHHHHHHHHCCCCC
CCCCCHHHHHHHHHHHHHHHHHHHHHHHHHHHHHHCCCCHHHHHHHHHHHHHHHC
CCCHHHHHHHHHHHHHHHHHHHHCCCEEECCCCCCCCCCCEEEEECCCCCCCCCCCE
EEEEEECCCCCCCCCCCCCCCCCCHHHHHHHHHCCCCCCCCCEEECCHHHHHHHHH
CCCCCCCCCCCCCCCCCCCCCCCCCCCCCCCCCCCCCCCCCCCCHHHHHHHHHH
HCCCCCCCCCCCCCCCCCCCHHHHHHHCCCCCCCEEEEEEECCCCEEEEEEECCHH
HHHHCCCCCCCCCCHHHHHHHHHHHHCCCEEEEECCCCCCCCCCCCCCCCCHHHHH
HCCCCEEEEEEEEECCCCCCCHHHHHHHHHHHCCCCEEEECCCCHHHHHHHHHHHCCCC
CCCCCEEEEECCCCCCCCCCCCCCCCCCCCCHHHHHCCCCCCCCCCCCCCCCCCCCC
CCCCCCEEECCCCCCCCCCCCCCCCCCCCCCCCCCCHHHHHHHHHHHHHHHHCC
CCCCCCCCCCCCCCCCCCCCCCCCCCCCCCCCCHHHHHHHHHCCCCCCCCCCCC
CCCCCCCCCCCCCCCCEEEECCCHHHCCCCCCCEEEEECCCCCCCCCHHHHHHHCC
CCCCCCCCCCCCCCHHHHHHHHHHHCCHHHHHHHHHHHCCCCCCCCHHHHHHHHHH
HHHCCCCCCCCCCCCCCCEEEECCCCCCCCCHHHHHHCCCCCCCCCCCCCCCCCCC
CCCCHHHHHHHHHHHHHHHHHHHHHHHHHHHHHHHHHHHHHHHHHHHHHHHHCCCCCHH
HHHHHHHHHHHHHHHHHCCCCCCCCCCCCCCCCCCCCHHHHHHHHHHHHHHHHHH
HHHHHHHHHHHHCCCCCCCCCCCCCCCCCCCHHHHHHHHHHHHHHHHHHHHHHHHCC
CCCCCCCCCCHHHHHHHHHHHHHHHHHHHHHCCCCCCCCCCCCCCCCCCCCCCCCCCH
HHHHHHHHHHHHHHHHHHHHHHHHHHHHHHHHHHHHHHHHHCCCCCCCCCCCCCCCCCC
CCCCCCCCCCCCCCCCCCCCCCC [Secondary structure predicted by PsiPred]

Target :
MGKGQNLAPATNSNGFHPHGAHPGPHHEEGHGHGAHGEEPNKSFTLLLSGQADDEDIASF
EGFHSPWWKKLLYYFVGLITAGFSFLMCKWSPRVHIFLSLSPCPLRDAQYVRIRLADGRVDLEK
VQEVAMTEPHYDSVPTLAADEEEGAGRGGLLDWQVQRTHKLLEYRCTRYFYVDGVAAAAAAV
AGGPAPGPGGATFTPVPALPKGFNEQLRSAAVTLATTGHAAAVEEAQEWDLGGRQLRYGTNE
MAIPVKSIPMLIFDEMWHPFYVFQYFSILIWIVGDAYYSYAVCIAVITWFSIISAAYEAHQNMKRL

AEIAHFECEVDVVRSGEVVRLPSSALVPGDLVVVAPGTLPCDMVLLRGECILDENMLTGESVPV
RKVEYNAVADGLAYQPDKCPGCTLFGGTVVAQARAPRAQKPLAMVCRTRFYSAKGQLLRSILF
PREPEESFISDSLRFICVMLAACMGLYIWAAVVLAQVGASPDRIVVRFFDMITIAVPPALPACLTI
ATVFSIGRLRKKGIYVTSPDRITLAGQLDVICFDKTGTLTEQGLDLQGIVPIVDGRLHNMVGQISL
LPTQLVELLASCHGLARMGESLVGDPLDQKLFVATHWDLIDERPSLDGAYGGAYGGSAGGA
GPQQTDIDGNVQGPGAGAAEAAQAEAEATGTGGGVQTYVRPPGAVHAYAIVKRFEFSAALQR
NLVVVRAPDNSVAVFAKGSPEAIRALADPGSIPADFDQLLGELTREGLRVLALAAGDASTVPDS
TLLGWTQAETEANVGLRLVGLAVMANPLRPDTADVISRLQHASIRTVMVTGDHLRTAVSVAHK
CNILPGQRPILLIDAADLPAPATASQQQPTPLPQVAAAAATQAPAGVEPALMPQSTPLPVLRPA
HAYSEASGPGPAGEPTPLGDPSIANPNWNGSLLTEEAEEAELRDAAALNTPYHGGASGLGDG
AGSSSEGGAGILSEYGPASQHAHVVVQAQYPGMAQPGGAAGKGAVPVDSGNGHHHTAVTA
AADAASAAAGPGLRLSVLDVEGNVTENAGSAAAYGMLARVVTGELECAVTGKGFNWLLASLD
AGLLFPVLQRAGVFARMSPDNKRDLMLLLGSGIDGVEGCPHLGLRAGFCGDGANDCGALKA
AHVGVSLCEAEASVAAPMTSKAQTIASMITVVAEGRCTLMATYQIFQFIIAYALVQAFETNLMYTY
ALNLGNYQYLIEDLFFTTVLAALMGFTEPRNKLSRSRPLTRVMSPPLMISTVLQCVVIVVFQLLSL
KLLQSQPTYVRFRGGPELHDTVAPENTTTYIVALAQFVVLALVFNKGMPHRSPLWTNLWLVAV
LVIQTAFVIYSLFSTDAFNMDVQQLVDKTSFNAVMDTEFRWKLFGLLVAMGITAFAAEYTSIGIVK
LLNWAQGRPGAGSVRGTKAGSPESKGSMPSTVPDAIRLPRQEQSGR

Match : ::G| : W| | Y| V : AG: :L| |W:P | L L PC L A| : I:| :L
VQ | |: RY:|| :F V L G V : G : D R: YG N ::IPVKS P:L|
DE :P|Y FQ FSI |W|: YY YA:CI :I: :SI : Y::::: : L |:: :: :V V R GE : SS LVPGD :V: |PCD :L:
GEC:::E: LTGES|PV K: A: |GL: TLF GT:| QARA : LA:V RT F :AKG L: SIL PR : :F : S||F|::| : :|
I|: |L : : ::IV|R ::||T|:VPPALPA:|T|:T:|: :RLR|:GI| P RI:L:G:L::|CFDKTGTLTE:GLD::G|VP| : |V
L L:: LA:CH:L:R| |: VGDP|D K| :T W :| :A|G :|: RF FS:ALQR
VVV :|:KGSPE | :L::P :|P:DF Q|L : T G RV:ALA: TVP: T: :E: L L:GL VM N L|P:T: VI: L:::
IR:VMVTGD:L:TAV:VA: C::: Q: |||| A: G|P:
L: L :E: : : A::G F: |: : LL VL :: VFARM:P|:K |L: L : :G:CGDGANDCGALKAA
VG|SL |AEASV::P:TS: :I : V| EGRC:L ::|:|F:|: Y:L:Q ::: ||YT NLG: Q|L :L :TT::A:LM: T P L:R
RP :|S P:| S :LQ V:|: QL : L :QP |V ENT::| :: Q|:|LA : :KG P R PL|TN: ::::L:| :: :| ::
DT F| L:GL:: | AF : : | L: : A:: R : :: P

Template: ....RLSGYCGSPWRVIGYHVVVWMMAGIP
LLLFRWKPLWGVRLRLRPCNLAHAETLVIEI.....QLFTVQVQ..................................RYYLFQ
GQRYIWI...................QAFYQVSLLDHGR....SCDDVHRSRHGL.......SLQDQMVRKAIYGPN
VISIPVKSYPQLLVDEALNPYYGFQAFSIALWLAD..YYWYALCIFLISSISICLSLYKTRKQSQTLR
DMVKLSMRVCVCR.GEEEWVDSSELVPGDCLVL....MPCDAALVAGECMVNESSLTGESIPVL
KT...ALPEGLG........HTLFCGTLILQARAYVGPHVLAVVTRTGFCTAKGGLVSSILHPRPINFKFY
KHSMKFVAALSVLALLGTIYSIFILYRNRVPLNEIVIRALNLVTVVVPPALPAAMTVCTLYAQSRLR
RQGIFCIHPLRINLGGKLQLVCFDKTGTLTEDGLDVMGVVPLKGQAFLPLVP..RLPVGPLLRAL
ATCHALSRLQDTPVGDPMDLKMVESTGW.VL..........SAFGT..
........VSVLHRFPFSSALQRMSVVVA.....PEAYVKGSPELVAGLCNPETVPTDFAQMLQSYTAAG

YRVVALASKPLPTVPSLEAAQ.TRDTVEG..DLSLLGLLVMRNLLKPQTTPVIQALRRTRIRAVMVT
GDNLQTAVTVARGCGMVAPQEHLIIVHAT...RGQPA
....SLEFLP
MES....................SRHLALSGPTFGIIVKHF..KLLPKVLVQGTVFARMAPEQKTELVCELQK.....
.........VGMCGDGANDCGALKAADVGISLSQAEASVVSPFTSSMASIECVPMVIREGRCSLDTS
FSVFKYMALYSLTQFISVLILYTINTNLGDLQFLAINLVITTTVAVLMSRTGPALVLGRVRPPGALLS
VPVLSSLLLQMVLVTGVQLGGYFLTLAQPWFVP...........NYENTVVFSLSSFQYLILAAAVSKGA
PFRRPLYTNVPFLVALALLSSVLVGLVLV...................TDTGFKLLLLGLVTLNFVGAFLESVLD
QCLPACLRRLR...RASKKRFKQLERELAEQPWPP................
